# Insect – fungus interactions: diversity, ecology, and applications

**DOI:** 10.3389/fcimb.2026.1943397

**Published:** 2026-10-20

**Authors:** Samantha C. Karunarathna, Anusha H. Ekanayaka, Namali T. de Silva, Shike Huang, Saowaluck Tibpromma, Sajid Ali, Martin Muthee Gakuubi, Jing-ya Yang, Entaj Tarafder, Dong-Qing Dai, Fuqiang Yu, Kalani K. Hapuarachchi, Leela M. Rizal

**Affiliations:** 1 Center for Yunnan Plateau Biological Resources Protection and Utilization & Yunnan International Joint Laboratory of Fungal Sustainable Utilization in South and Southeast Asia, College of Biology and Food Engineering, Qujing Normal University, Qujing, China; 2 Key Laboratory of Yunnan Provincial Department of Education of the Deep-Time Evolution on Biodiversity from the Origin of the Pearl River, Qujing Normal University, Qujing, Yunnan, China; 3 National Institute of Fundamental Studies, Kandy, Sri Lanka; 4 Department of Urban Bioresources, Faculty of Urban and Aquatic Bioresources, University of Sri Jayewardenepura, Nugegoda, Sri Lanka; 5 College of Resources and Environment, Zunyi Normal University, Zunyi, Guizhou, China; 6 Engineering Research Center of Southwest Bio-Pharmaceutical Resources, Ministry of Education, Guizhou University, Guiyang, Guizhou, China; 7 Department of Biological Sciences, College of Science, King Faisal University, Al-Ahsa, Saudi Arabia; 8 Department of Agriculture, Earth and Environmental Sciences, Faculty of Science, Mwenge Catholic University, Moshi, Kilimanjaro, Tanzania; 9 College of Agronomy and Biological Science, Yuxi Normal University, Yuxi, Yunnan, China; 10 Germplasm Bank of Wild Species & Yunnan International Joint Laboratory of Fungal Sustainable Utilization in South and Southeast Asia, Kunming Institute of Botany, Chinese Academy of Sciences, Kunming, China; 11 College of Biodiversity Conservation, Southwest Forestry University, Kunming, China; 12 Commonwealth Scientific and Industrial Research Organisation (CSIRO) Agriculture and Food, Canberra, ACT, Australia

**Keywords:** coevolution, entomopathogenic fungi, fungal bio-control, mutualism, plant pests

## Abstract

The coevolution of insects and fungi over more than 400 million years has produced a remarkable diversity of ecological associations, spanning obligate mutualisms to antagonistic parasitism. This systematic review synthesizes current knowledge of insect–fungus interactions by integrating ecological, evolutionary, and applied perspectives through a structured literature synthesis and network analysis of documented associations. Multiple independent evolutionary origins of insect–fungus mutualism are identified, with Coleoptera, Hymenoptera, and Blattodea most frequently participating in mutualistic associations. Nutritional mutualisms, such as fungus farming by ambrosia beetles, termites, and leaf-cutter ants, are the most ecologically significant interactions. These are characterized by specialized morphological adaptations (mycangia, fungus combs), enzymatic capabilities for lignocellulose degradation, and co-evolutionary dynamics that have shaped both partners’ genomes. Commensal associations, particularly gut-dwelling fungi in aquatic insects, display context-dependent outcomes that may shift toward mutualism under certain environmental conditions. Entomopathogenic fungi, distributed across diverse lineages including Sordariomycetes and Entomophthoromycetes, act as major mortality agents in insect populations and employ sophisticated mechanisms of host manipulation, such as behavioral modification and immune suppression. In addition to their ecological roles, insect–fungus associations have substantial practical applications. Entomopathogenic fungi offer eco-friendly alternatives to synthetic pesticides in biological control. Fungal symbionts contribute to bioremediation through lignocellulolytic and detoxification enzymes. Medicinal fungi, such as *Cordyceps* species, produce bioactive compounds with pharmaceutical potential. Critical knowledge gaps remain, including the undercharacterized functional diversity of insect-associated fungal communities, the molecular mechanisms underlying host specificity, and the impacts of climate change on interaction outcomes. By integrating network analysis with comprehensive literature synthesis, this review establishes a framework for understanding the ecological and evolutionary significance of insect–fungus associations and highlights their translational potential for sustainable agriculture, biotechnology, and medicine.

## 1 Introduction

Insects are the most diverse animal lineage, with over one million formally described species and an estimated 5.5 million species, accounting for approximately 75-80% of all described animal species (Hulcr and Dunn, 2011; Stork et al., 2015; Li and Wiens, 2023; Slade and Ong, 2023; Hailay Gebremariam, 2024; Chesters, 2025; Agregán et al., 2026). This massive diversity of insects underscores their extraordinary evolutionary success and ecological dominance with an evolutionary history of more than 400 million years (Gebremariam, 2024). The vast diversity of insects correlates with an equally complex array of insect ecological interactions with other organisms, particularly with microorganisms such as fungi. These interactions have over the years played an important role in shaping insect biology, adaptation, and ecosystem functioning. Among these interactions, insect-fungus associations are particularly significant, influencing many ecological processes ranging from nutrient cycling to forest health and population dynamics. Insects and fungi represent key components of ecological interactions and functional biodiversity, with their associations shaping many ecosystem processes (Malacrinò, 2017). For example, community resistance to invasive forest insect–fungus mutualisms can arise through antagonistic interactions with native competitors and natural enemies (Thompson et al., 2019). The influence of biosecurity on the diversity and management of a 120-year-old joint insect-fungus pest invasion in Australia and New Zealand reveals how long-term invasions affect the genetic structure and diversity of associated fungal symbionts (Eshetu et al., 2025). Furthermore, insect symbionts act as hidden players in insect–plant interactions, often mediating outcomes in ways that are not apparent when examined solely at the insect-plant interface (Frago et al., 2012).

Fungi, the second most species-rich eukaryotic kingdom are estimated to comprise between 2.2 and 3.8 million species (Hawksworth and Lücking, 2017; Cunningham et al., 2024; Hyde et al., 2024; Wang et al., 2026). Their prolonged coexistence with insects, spanning over 400 million years, has given rise to a wide spectrum of interactions, which can be categorized as mutualism (beneficial to both), commensalism (beneficial to one, neutral to the other), parasitism or predation (beneficial to one, detrimental to the other), and competition (detrimental to both) (Biedermann and Vega, 2020; El-Metwally et al., 2023; Musa et al., 2026; Arun Kumar, 2026). Among these, mutualist interactions are particularly prevalent in insect–fungal associations. Some well-known examples include fungi cultivated by ambrosia beetles, mushroom gardens maintained by leaf-cutter ants and termites, as classically described by Batra and Batra (1967), and fungi that associate with scale insects. These relationships involve structural, biochemical, and behavioral adaptations in both partners (Green et al., 2025; Lehenberger et al., 2025; Pyszko et al., 2025; Schmidt et al., 2025; O’Brien et al., 2026). Plant symbiotic fungi also mediate insect herbivore interactions in a multitrophic context, with cascading effects on host plant growth, defense, and fitness (Hartley and Gange, 2009). Microbial brokers of insect-plant interactions, including gut symbionts and plant-associated microbes, play critical roles in mediating nutritional exchange, influencing dietary nitrogen quality, and shaping the outcomes of herbivory (Douglas, 1992). Three-way interactions among plants, pathogenic fungi, and herbivorous insects involve complex dynamics in which the presence of one species can alter the outcome of interactions among the other two (Hatcher, 1995). Endophytic fungi, in particular, mediate complex tripartite interactions among vascular plants, insect herbivores, and themselves, often altering plant chemistry and insect behavior (Raman et al., 2012). The study of insect–fungal interactions hold substantial application potential. Entomopathogenic fungi are increasingly recognized as effective and sustainable biocontrol agents against agricultural insect pests (Kinyanjui et al., 2025; Schläppi et al., 2025; Jiaqin and Nicolás, 2025; Mussa et al., 2026; Hendra et al., 2026). Fungi also show promise in protecting honeybees during commercial beekeeping operations, a critical need given the global pollinator declines (Bava et al., 2022; Güneşdoğdu et al., 2025). Additionally, insect–fungal systems contribute to biodegradation of complex substrates and offer potential for bioremediation (Barcoto and Rodrigues, 2022; Asemoloye et al., 2025; Yi et al., 2025). The biology and pathology of interactions between fungi and invertebrates span a wide spectrum from beneficial symbioses to lethal infections, with important implications for veterinary and agricultural sciences (Williams, 2003). Fungal endophyte-plant-insect tripartite associations are an understudied area that warrants attention, as these interactions can significantly influence pest management and crop protection (Suryanarayanan, 2023). The economically valuable genus *Cordyceps*, which parasitizes insects, further exemplifies the significance of these associations (Turk et al., 2022; Zhang et al., 2026a). A comprehensive global review of mycophagy reveals that invertebrate–fungal interactions are more widespread than previously recognized, with significant implications for nutrient cycling and ecosystem functioning (Santamaria et al., 2023).

Recent advances in meta-omics approaches have greatly revolutionized the study of insect-microorganism interactions. Omics technologies have enabled culture-independent analyses of microbial communities, enabling comprehensive identification and functional characterization of mutualistic, commensal, and pathogenic associations across diverse insect taxa (Malacrinò, 2018). Beyond taxonomic profiling, such approaches allow for critical assessment of gene expression dynamics, metabolic pathways, and chemical signaling systems that underpin insect-microbe interactions. As a result, the molecular mechanisms underlying many insect–fungi interactions have been extensively characterized, revealing complex genomic adaptations on both sides (Wang and Wang, 2017; Dubovskiy, 2021).

This review proposes that insect–fungus associations exhibit patterns of ecological convergence driven by shared selective pressures. It is hypothesized that the repeated evolution of nutritional mutualisms, entomopathogenicity, and context-dependent symbioses across phylogenetically diverse fungal and insect lineages reflects convergent responses to common ecological challenges, such as exploiting nutrient-limited substrates (for example, lignocellulose in plant biomass) and the need for dispersal in sessile organisms. These convergent selective pressures are predicted to have consistently favored expansions in specific carbohydrate-active enzyme families (including AA4, CE4, and GH3) and the evolution of specialized morphological structures (such as mycangia and fungus combs), regardless of the phylogenetic distance between interacting partners. By synthesizing ecological, evolutionary, and genomic evidence, this review tests this hypothesis and establishes a unified framework for understanding the drivers and consequences of insect–fungus associations.

Although numerous studies have examined specific insect–fungal interactions (Biedermann and Vega, 2020; Ma et al., 2024; Bachelot et al., 2025), a comprehensive synthesis integrating ecological, evolutionary, and applied perspectives is currently lacking. This study aims to fill this gap by synthesizing current knowledge of insect–fungal associations, with emphasis on the diversity of fungal partners, and the ecological functions of their interactions.

## 2 Methodology

### 2.1 Literature review

The following search terms and Boolean operators were used: (“insect-fungus interaction” OR “entomopathogen*” OR “entomopathogenic fungi” OR “fungus-farming insect” OR “fungus-growing insect” OR “insect-fungus mutualism” OR “insect-fungal mutualism” OR “insect-fungus symbiosis” OR “insect-fungal symbiosis” OR “insect-fungus commensal” OR “insect mycophagy” OR “fungivory” OR “fungal biocontrol”). The following electronic databases were searched: Web of Science Core Collection, Scopus, PubMed, and Google Scholar. The last search date was 28 April 2026.

Initial database searches identified 1, 247 records. After duplicate removal, 1, 102 records remained. Titles and abstracts were screened, resulting in 412 full-text articles assessed for eligibility. We excluded 370 articles based on criteria such as no documented insect involvement, insufficient evidence of ecological interactions, or uncertain taxonomic identification. Ultimately, 42 studies fulfilled all inclusion criteria and were incorporated into the final qualitative synthesis. The PRISMA flowchart (**Figure 1**) summarizes the screening process.

**Figure 1:**
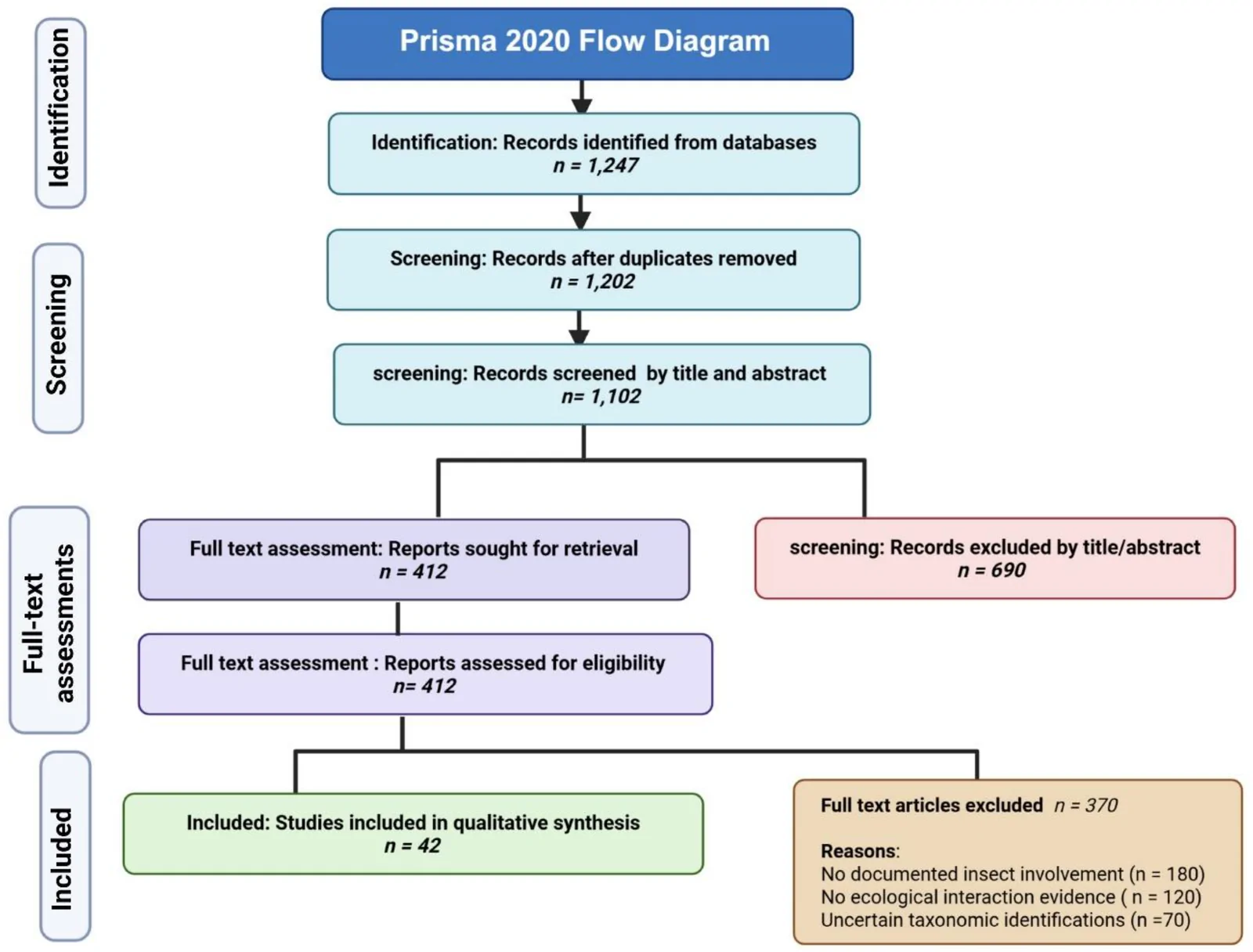
PRISMA 2020 flow diagram illustrating the systematic literature screening process. Initial database searches (Web of Science, Scopus, PubMed, Google Scholar) yielded 1, 247 records. After duplicate removal (1, 102 records), title/abstract screening, and full-text assessment (412 articles), 42 studies met the inclusion criteria and were included in the final qualitative synthesis (Adapted from Page et al. (2021) and created with BioRender).

#### 2.1.1 Inclusion criteria

Studies were included if they: (1) documented insect-fungal interactions with identified fungal species (at least morphological identification) and insect order; (2) provided explicit evidence for interaction type (mutualism, commensalism, parasitism, or mycophagy); and (3) were published in English.

#### 2.1.2 Exclusion criteria

Studies were excluded if they: (1) focused exclusively on plant pathogens without documented insect involvement; (2) described fungal isolates from insects without evidence of ecological interaction; (3) were conference abstracts without sufficient methodological detail; or (4) had uncertain taxonomic identification.

#### 2.1.3 Screening procedure

Records were screened by title, then by abstract, and finally by full text against the inclusion and exclusion criteria. Studies that met all criteria were included in the final analysis. Data extraction was performed independently by two reviewers, with discrepancies resolved through consensus.

#### 2.1.4 Data extraction

For each included study, the following data were extracted: fungal species, fungal class (according to MycoBank/Index Fungorum), insect order (according to Catalogue of Life), interaction type (as reported by original authors), and context of interaction. In addition, bibliographies from key publications, including relevant review articles and book chapters, were manually screened to identify additional studies not captured by the database searches. Fungal taxonomy follows MycoBank and Index Fungorum; insect taxonomy follows the Catalogue of Life.

**Table 1** provides an overview of Fungi – insect interactions.

**Table 1:** Fungi – insect interactions.

Association	Fungal species (class)	Insect order	Context of interaction	Reference(s)
Mutualism	*Amylostereum arelatum* (Agaricomycetes)	Hymenoptera	Female wasps inoculate fungus into wood during oviposition; the fungus decays the sapwood; larvae feed on it.	Slippers et al. (2003)
	*Epichloë typhina* (Sordariomycetes)	Diptera	Female flies transfer spores among self-incompatible mycelia during oviposition; larvae feed on resulting perithecia.	Bultman and Leuchtmann (2008)
	*Ophiostoma ulmi* (Sordariomycetes)	Coleoptera	Beetles carry spores externally and infect new trees; fungus degrades xylem; beetles colonize dead xylem.	Santini and Faccoli (2015)
	*Entomocorticium* spp. (Agaricomycetes)	Coleoptera	Beetle mycangia carry fungus; inoculated galleries serve as a larval food source.	Araújo et al. (2021); Harrington et al. (2021)
	*Septobasidium* spp. (Pucciniomycetes)	Hemiptera	Scale insects live in fungal thallus; the fungus infects some individuals and gains nutrients and dispersal.	Murrin (1996)
	*Botryosphaeria dothidea* (Dothideomycetes)	Diptera	The fungus is essential for gall formation; larvae feed on the fungus within the gall.	Heath and Stireman (2010)
	*Cladosporium cladosporioides* (Dothideomycetes)	Hemiptera	Associated with gall-forming insects; provides nutritional benefits.	Lawson et al. (2014)
	*Cladosporium sphaerospermum* (Dothideomycetes)	Hemiptera	Associated with adelgid galls; possible nutritional mutualism.	Lasota et al. (1983)
	*Cladosporium oxysporum* (Dothideomycetes)	Coleoptera	Larvae feed on fungus in leaf rolls.	Carrion and Bonet (2004)
	*Termitomyces* spp. (Agaricomycetes)	Blattodea	Termites cultivate fungus; fungus degrades plant material for larvae.	Aanen et al. (2002)
	*Leucocoprinus*, *Leucoagaricus gongylophorus* (Agaricomycetes)	Hymenoptera	Ants cultivate fungus; fungus converts plant material into food for larvae.	Chapela et al. (1994); Urrea-Valencia et al. (2023)
	*Cylindrobasidium ipidophilum*, *Phlebiopsis gigantea* (Agaricomycetes)	Coleoptera	Fungi are carried in mycangia; larvae feed on the fungus in galleries.	Harrington et al. (2021)
	*Ambrosiella*, *Raffaelea*, *Geosmithia* spp. (Sordariomycetes)	Coleoptera	Fungi grow in beetle galleries; larvae feed on them.	Brown (1954); Kajimura and Hijii (1992); Joseph and Keyhani (2021)
	*Symbiotaphrina* (Xylonomycetes), *Candida* (Saccharomycetes), *Taphrina* spp. (Taphrinomycetes)	Coleoptera	Fungi in mycetoma improve larval development by detoxifying the diet.	Shen and Dowd (1991); Murrin (1996)
	*Cryptococcus* (Tremellomycetes), *Rhodotorula* (Microbotryomycetes), *Debaryomyces* (Saccharomycetes), *Trametes* (Agaricomycetes), *Penicillium* spp. (Eurotiomycetes)	Hemiptera	Gut fungi involved in nitrogen recycling.	Vera-Ponce de León et al. (2016)
	*Cladosporium* spp. (Dothideomycetes)	Trichoptera	Improves the digestion of cellulose and xylan diets.	Souza et al. (2022)
	*Cladosporium* spp.	Diptera	Assists in sugar assimilation in nectar diets.	Guégan et al. (2020)
	*Yarrowia* spp. (Saccharomycetes)	Coleoptera	Preserves carcass and enables larval feeding.	Shukla et al. (2018)
	*Claviceps purpureum* (Sordariomycetes)	Diptera, Coleoptera	Attracts insects for spore dispersal via sugary exudate.	Lemon (1992)
	*Metschnikowia* (Saccharomycetes), *Cryptococcus* (Tremellomycetes), *Aureobasidium pullulans* (Dothideomycetes)	Hymenoptera	Influence nectar scent and improve bee fitness.	Jacquemyn et al. (2021)
	*Phallus* spp. (Agaricomycetes)	Diptera	Attract flies for spore dispersal via odor and spores.	Tuno (1998)
	*Puccinia monoica* (Pucciniomycetes)	Lepidoptera	Creates pseudoflowers to attract pollinators.	Roy (1996)
	*Agaricus* spp. (Agaricomycetes)	Diptera	Larvae feed on fungi; spores are dispersed via feces.	Bunyard (2007)
Commensalism	*Grosmannia clavigera*, *Ophiostoma montium* (Sordariomycetes)	Hymenoptera	Wasps use fungal volatiles to locate hosts; the fungus is unaffected.	Adams and Six (2008)
	*Harpella, Stachylina* spp. (Harpellomycetes)	Multiple orders	Fungi attach to the gut lining; they gain nutrients without harming the host.	Murrin (1996); Alencar et al. (2003)
Parasitism	*Laboulbenia* spp. (Laboulbeniomycetes)	Hemiptera, Coleoptera	Ectoparasitic; extracts nutrients from the host.	De Kesel et al. (2020); Kaishian (2020)
	*Ascosphaeria apis* (Eurotiomycetes)	Hymenoptera	Causes chalkbrood disease in bees.	Chen et al. (2018)
	*Ophiocordyceps unilateralis* (Sordariomycetes)	Hymenoptera	Manipulates ant behavior for spore dispersal.	Andersen et al. (2009)
	*Ophiocordyceps sinensis* (Sordariomycetes)	Lepidoptera	Mummifies larvae; fruiting body emerges from the host.	Hu et al. (2013)
	*Smittium morbosum* (Harpellomycetes)	Diptera	Kills larvae by preventing molting.	Sweeney (1981)
	*Metarhizium anisopliae* (Sordariomycetes)	Coleoptera, Hymenoptera, Hemiptera Lepidoptera	Causes green muscardine disease.	Wang et al. (2012a), Rizal et al. (2022); Rizal (2023), Rizal et al. (2024)
	*Beauveria bassiana* (Sordariomycetes)	Blattodea Thysanoptera Homoptera Coleoptera Lepidoptera	Generalist pathogen; used as biocontrol.	Khun et al. (2021); Rizal et al. (2022); Rizal (2023); Rizal et al. (2024), Rizal et al. (2026)
	*Isaria fumosorosea (Sordariomycetes)*	Multiple orders	Produces toxins that kill the host.	Ali et al. (2010)
	*Akanthomyces lecanii (Sordariomycete)*	Hemiptera, Thysanoptera	Infects and proliferates inside an insect.	Chang et al., 2024
	*Cladosporium cladosporioides (Dothideomycetes)*	Hemiptera	Overgrows and disrupts insect protective layers.	Kishore et al. (2019)
	*Hirsutella thompsonii (Sordariomycetes)*	Acarina	Produces toxin harmful to mites.	Herrero-Galán et al. (2013)
	*Nomuraea rileyi (Sordariomycetes)*	Lepidoptera	Kills the host via nutrient depletion and toxins.	Padanad and Krishnaraj (2009)
	*Massospora cicadina (Entomophthoromycetes)*	Hemiptera	Alters behavior for spore spread.	Cooley et al. (2018)
	*Entomophthora muscae*	Diptera	Causes summit disease in flies.	Elya and De Fine Licht (2021)
	*Entomophaga maimaiga*	Lepidoptera	Kills gypsy moth larvae.	Hajek (1999)
	*Pandora neoaphidis*, *Entomophthora planchoniana*	Hemiptera	Rapid infection and death in aphids.	Ben Fekih et al. (2013)
	*Erynia neoaphidis* ((Entomophthoromycetes_	Hemiptera	Sporulates from aphid cadavers.	Hemmati et al. (2001)
	*Entomophaga* spp. (Entomophthoromycetes)	Orthoptera	Infects grasshoppers; spreads via spores.	Bidochka et al. (1995)
	*Tolypocladium inflatum* (*Sordariomycetes*)	Coleoptera	Produces cyclosporin; parasitic.	Yang et al. (2018)
	*Zoophthora radicans* (Entomophthoromycetes)	Multiple orders	Infects and kills insect hosts.	Batta et al. (2011)
	*Batkoa major (Entomophthoromycetes)*	Hemiptera	Infects and kills planthoppers.	Hajek et al. (2022)
	*Strongwellsea castrans* (Entomophthoromycetes)	Diptera	Discharges spores while the host is alive.	Eilenberg and Jensen (2018)
	*Trichoderma atroviride* (Sordariomycetes)	Lepidoptera, Hemiptera	Retards larval growth; attracts parasitoids.	Coppola et al. (2019)
	*Cordyceps* (Sordariomycetes)	Lepidoptera	parasitizes diamondback moth, proliferates outside the larvae and adults, and kills.	Rizal et al. (2024)
Mycophagy	*Agaricus*, *Pleurotus*, *Russula*, *Amanita* spp. (Agaricomycetes)	Diptera, Coleoptera	Insects feed on fungal tissues and spores.	Murrin (1996); Oyebamiji et al. (2018); Sharma et al. (2019)

Interaction types (Mutualism, Commensalism, Parasitism) are classified as reported by the original authors, based on fitness outcomes. Mycophagy is treated as a feeding behavior that can occur within any of these categories. This review uses the term ‘symbiosis’ as a broad ecological umbrella term encompassing close, long-term associations between different species, and does not treat it as a separate interaction category.

Detailed field studies on interactions between mycophagous Drosophilidae (Diptera) and macroscopic fungi (Basidiomycota) have revealed specialized breeding networks and stronger interactions among phylogenetically related species (Duarte et al., 2024). New host records for three scuttle flies, *Megaselia flava*, *M. kanekoi*, and *M. gotoi* (Diptera: Phoridae), on the poisonous fungus *Amanita ibotengutake* demonstrate specialized insect-fungus associations that may be strongly involved in insect-fungus interactions and warrant further study (Yamashita et al., 2005). The diversity of symbiotic interactions between species of macrofungi and insects in temperate forests reveals nutritional interactions that contribute to fungal spore dispersal and insect development (Garza-Ocañas et al., 2023). Additionally, a comprehensive summary of mycophagy in insects provides a tabular review of insect-fungus interactions in which insects exploit fungal resources (Hammond et al., 1989). The effects of fungivory by two specialist ciid beetles (*Octotemnus glabriculus* and *Cis boleti*) on the reproductive fitness of their host fungus *Trametes versicolor* demonstrate that insect grazing can significantly reduce fungal spore production and overall reproductive success, with implications for fungal population dynamics (Guevara et al., 2000). Moreover, insect parasitism of edible ectomycorrhizal mushrooms represents specialized interactions that can significantly impact mushroom production and harvest (Sitta and Süss, 2013).

### 2.2 Data visualization (Sankey diagram)

To visualize the network of insect–fungal associations, a Sankey diagram was constructed from the dataset presented in **Table 1**. For each interaction, three attributes were extracted: (1) fungal class (according to MycoBank/Index Fungorum), (2) interaction type (mutualism, commensalism, parasitism, or mycophagy as reported by the original authors), and (3) insect order (according to the Catalogue of Life). The data were organized into a three-column table and imported into an online Sankey diagram tool (SankeyMATIC, https://sankeymatic.com/). Ribbon widths were scaled proportionally to the frequency of associations between each fungal class, interaction type, and insect order. The resulting diagram (**Figure 1**) was exported as a high-resolution image. This approach enables visualization of the relative strength of associations across the three categorical variables.

In this review, interaction types are classified according to the fitness outcomes for the interacting partners. Mutualism refers to interactions in which both partners benefit (+/+). Commensalism describes interactions where one partner benefits and the other remains unaffected (+/0). Parasitism involves one partner benefiting at the expense of the other (+/-). Mycophagy, or fungus-feeding, is considered a feeding behavior rather than a distinct interaction type and may occur within any of the three outcome-based categories. The term symbiosis is applied as a broad ecological concept encompassing all close, long-term associations between different species, including mutualism, commensalism, and parasitism. These definitions provide mutually exclusive categorical criteria for the network analysis.

#### 2.2.1 Data transparency

The complete dataset underlying the Sankey diagram is presented in **Table 1**.

### 2.3 Data synthesis and qualitative analysis

The Sankey diagram was selected as the primary visualization tool because it effectively displays the flow of interactions across three categorical variables (fungal class → interaction type → insect order) simultaneously, revealing patterns that are not apparent from tabular data alone. Ribbon widths are scaled proportionally to interaction frequencies, allowing visual identification of dominant associations. Because this is a systematic review with qualitative synthesis rather than a meta-analysis, we do not perform statistical hypothesis testing. Instead, we report descriptive patterns and explicitly acknowledge that observed frequencies reflect both ecological patterns and biases in sampling/research effort. Patterns described in the Results section are based on visual interpretation of the Sankey diagram flows and qualitative assessment of the compiled dataset.

The Sankey diagram (**Figure 2**) serves as a visualization tool designed to facilitate rapid comprehension of the relative frequencies of documented interactions within the compiled literature dataset, rather than providing a quantitative inference of ecological abundance. Ribbon widths represent frequencies reported in the reviewed studies and reflect sampling biases, research effort, and taxonomic and geographic reporting biases. For instance, the apparent dominance of Sordariomycetes (24 records) over *Harpellomycetes* (3 records) likely reflects intensive research on economically significant entomopathogens rather than true ecological prevalence. Consequently, the diagram should be regarded as a descriptive summary of the compiled literature, not as a statistical representation of global ecological patterns. Formal meta-analytic controls were not implemented, as this work constitutes a qualitative systematic review with network visualization rather than a quantitative meta-analysis

**Figure 2:**
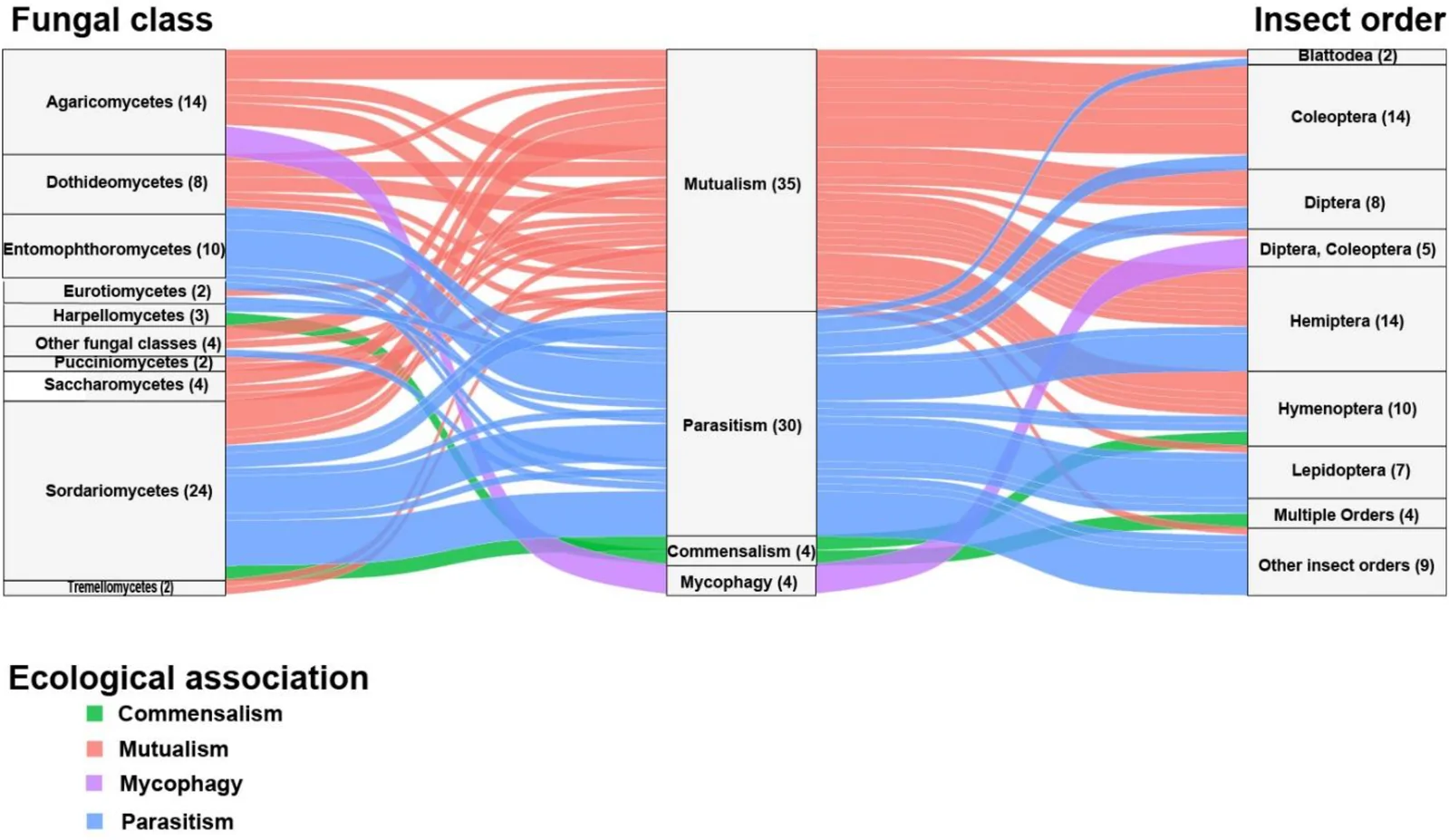
Sankey diagram visualizing the relative frequency of documented interactions among fungal classes (left column), interaction types (middle column), and insect orders (right column) within the compiled literature dataset. Ribbon width is proportional to the number of recorded associations in the reviewed studies (see **Supplementary Table S1** for the complete dataset). Important: The diagram reflects frequencies in the compiled literature, not global ecological abundance. The patterns are influenced by sampling biases, research effort, and taxonomic and geographic reporting biases. Interaction types are classified as Mutualism, Commensalism, and Parasitism, with mycophagy treated as a feeding behavior occurring within these categories. The diagram was constructed using SankeyMATIC (https://sankeymatic.com/) and data extracted from the literature, following the methodology described in Section 2.”.

## 3 Results

### 3.1 Fungal classes

The Sankey diagram (**Figure 2**) visualizes the relative frequency of associations for each fungal class.

Minor contributions are observed for Xylonomycetes, Tremellomycetes, Taphrinomycetes, Pucciniomycetes, Laboulbeniomycetes, and *Cordyceps*, which may reflect either ecological rarity or under-reporting in the literature.

Among fungal classes, Sordariomycetes (24 records), Agaricomycetes (14 records), and Entomophthoromycetes (10 records) contributed the largest interaction flows. Sordariomycetes exhibited the broadest ecological distribution, contributing to all interaction categories and associating with a wide range of insect orders, suggesting high ecological versatility. This class includes several well-known entomopathogenic and symbiotic genera, including *Metarhizium, Beauveria*, and *Ophiocordyceps*.

Entomophthoromycetes were strongly associated with parasitic interactions with Hemiptera and Diptera. In contrast, Agaricomycetes were primarily associated with mutualistic and commensal interactions, particularly with Coleoptera. Dothideomycetes and Saccharomycetes contributed intermediate numbers of interactions, whereas Eurotiomycetes, Harpellomycetes, Pucciniomycetes, and several minor fungal classes were comparatively infrequent in the compiled dataset.

The Sankey diagram reveals distinct association patterns between fungal classes and insect orders. Sordariomycetes show the broadest ecological distribution, contributing to all interaction categories and associating with a wide range of insect orders. Entomophthoromycetes are strongly associated with parasitism, particularly of Hemiptera and Diptera. Agaricomycetes are primarily associated with mutualistic and commensal interactions, particularly with Coleoptera. Dothideomycetes and Saccharomycetes contribute intermediate numbers of interactions, whereas Eurotiomycetes, Harpellomycetes, Pucciniomycetes, and several minor fungal classes are comparatively infrequent in the compiled dataset.

### 3.2 Interaction types

The distribution of interaction types is shown in **Figure 1**. Parasitism is the most prevalent interaction type, receiving the largest inflow from fungal classes and outflow to insect orders. This pattern indicates that pathogenic associations dominate the documented insect–fungus relationships. Mutualism is the second-largest category, followed by commensalism. Mycophagy, treated as a feeding behavior, is comparatively minor in the compiled dataset, which may reflect either lower ecological frequency or historical research bias toward parasitic and mutualistic systems. Throughout this review, ‘symbiosis’ is used as an umbrella term encompassing mutualism, commensalism, and parasitism, consistent with its broad ecological definition, and is not treated as a separate interaction category.

### 3.3 Insect orders

**Figure 1** also reveals which insect orders are most frequently associated with fungi. Coleoptera (beetles) receive the broadest and largest flows, participating in parasitism, mutualism, and commensalism. This indicates that beetles are involved in the widest range of fungal interactions in the dataset. Hemiptera (true bugs) and Diptera (flies) are also major endpoints, primarily linked to parasitism. This aligns with the high susceptibility of sap-feeding insects and flies to fungal pathogens. Hymenoptera (ants, bees, wasps), Lepidoptera (butterflies and moths), and Orthoptera (grasshoppers and crickets) show moderate associations. Smaller flows are observed for Thysanoptera (thrips), Trichoptera (caddisflies), Plecoptera (stoneflies), Blattodea (cockroaches and termites), and Acarina (mites).

### 3.4 Key ecological patterns

Several key ecological patterns emerge from **Figure 1**.

#### 3.4.1 Specialised parasitism

Entomophthoromycetes show a highly concentrated flow toward parasitism of Diptera and Hemiptera, consistent with their biology as obligate insect pathogens (e.g., *Entomophthora muscae* infecting houseflies).

#### 3.4.2 Mutualism with beetles

Agaricomycetes are strongly associated with mutualism, particularly with Coleoptera, reflecting well-documented fungus-farming symbioses such as bark beetles cultivating ambrosia fungi.

#### 3.4.3 Generalist interactions

Sordariomycetes exhibit the greatest ecological versatility, contributing to multiple interaction types across diverse insect orders, which aligns with the broad host ranges of genera such as *Metarhizium*, *Beauveria*, and *Ophiocordyceps*.

### 3.5 Caveats

The Sankey diagram (**Figure 1**) is a descriptive visualization of the compiled literature dataset, not a quantitative ecological inference. The ribbon widths represent frequencies of documented associations in the reviewed studies, which are subject to significant biases. These include research effort biases (e.g., overrepresentation of economically important groups such as entomopathogens and bark beetle symbionts), taxonomic biases (e.g., underrepresentation of understudied groups such as Harpellomycetes and Laboulbeniomycetes), and geographic biases (e.g., overrepresentation of temperate regions). Therefore, the diagram should not be interpreted as reflecting true ecological abundance or prevalence in nature.

The diagram reflects the compiled dataset rather than global ecological patterns. Observed flow widths may be influenced by sampling bias, historical research effort, and taxonomic reporting biases. Therefore, the patterns described above should be interpreted as trends within the reviewed literature rather than definitive ecological rankings. Representative examples of insect–fungal relationships are illustrated in **Figure 3**.

**Figure 3:**
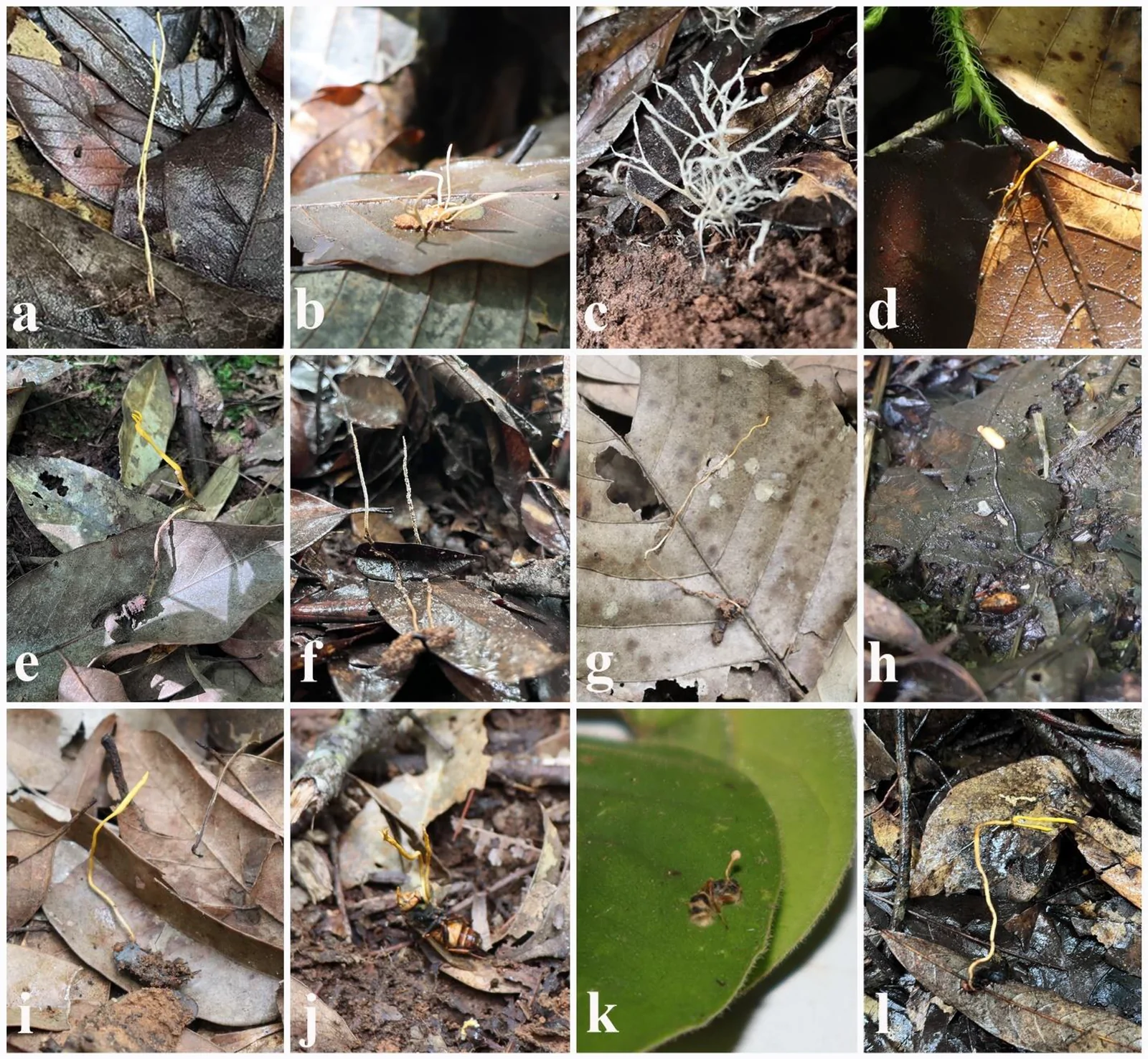
Illustrative field photographs of entomopathogenic fungi on arthropod hosts in leaf-litter habitats. Photographs represent original field observations by the author, Shike Huang, during surveys conducted in Yunnan and Guizhou provinces from August to October 2024. **(a–l)** Representative field images of infected arthropods (predominantly ants) colonized by cordycipitoid fungi. **(a)** Slender, pale stroma emerging from a host embedded in leaf litter. **(b)** Ant cadaver attached to the leaf surface bearing multiple filamentous stromata. **(c)** White, branched mycelial growth on the substrate. **(d)** Stromata emerging from a host attached to the underside of a leaf. **(e)** Long, curved stroma arising from an infected host on leaf litter. **(f)** Multiple elongated stromata emerging from partially buried host remains. **(g)** Thin, wiry stroma extending across a decaying leaf surface. **(h)** Early stage of stromatal development from a decomposed host. **(i)** Single elongated stroma emerging from a host in dry leaf litter. **(j)** Prominent clavate stroma emerging from an insect cadaver. **(k)** Infected ant attached to the leaf surface with a stroma arising from the head region, consistent with the characteristic ‘death grip’ behaviour. **(l)** Long, sinuous stroma extending from a host embedded in a moist substrate. These images are provided for illustrative purposes only to give readers a visual reference for entomopathogenic fungi in their natural habitats. They are not part of the systematic analysis and are not intended to represent quantitative patterns, prevalence, or taxonomic confirmation. Taxonomic assignments are preliminary and based on field macromorphological characteristics; species-level identification was not attempted. The images are opportunistic field observations and should not be interpreted as quantitative data. (**a–e, h–k** collected from Pu’er County, Yunnan; photographs by Shike Huang.).

### 3.6 Dataset characteristics and limitations

The dataset underlying this analysis comprises documented interactions from the published literature meeting our inclusion criteria. Several limitations should be noted. First, the dataset represents a fraction of the true diversity of insect–fungus associations, which likely numbers in the thousands. Second, taxonomic and geographic biases in the literature may skew observed patterns toward well-studied systems (e.g., bark beetles, fungus-farming ants, and economically important entomopathogens). Third, mycophagy was treated as a feeding behavior rather than an interaction type, potentially underrepresenting the ecological significance of fungivory. Fourth, the dataset reflects publication bias, with positive and significant results more likely to be published than negative or neutral findings.

## 4 Major insect–fungal interactions

Symbiotic relationships are ubiquitous in nature and appear to be fundamental to many biological systems. These interactions occur between distantly related organisms (e.g., bacteria and fungi) as well as among closely related species, like ants (Krishnan et al., 2024; Richards and Moran, 2024; Becks et al., 2025). The extent of interaction in these relationships varies, spanning from highly integrated obligate and stable associations such as those found in lichens (where the mycobiont (fungus) is intimately associated with the photobiont (algae/cyanobacteria)) to more loosely connected partnerships between separate organisms (Qiu et al., 2025; Rooy et al., 2025).

Insect–fungus symbioses are prevalent and can vary from mutualistic to antagonistic interactions (Nicoletti et al., 2024). Different groups of fungi exhibit diverse strategies for exploiting their environments, which in turn influence the evolution of both symbiotic partners (Mendes-Pereira et al., 2022; Assis et al., 2025; Elliot et al., 2025). The spectrum of insect–fungus interactions can be broadly categorized into mutualism, commensalism, and parasitism, with symbiosis serving as an umbrella term for close associations. Mycophagy, or fungus-feeding, is a behavioral strategy that can occur within any of these categories. **Figure 4** illustrates these interaction types and their defining characteristics.

**Figure 4:**
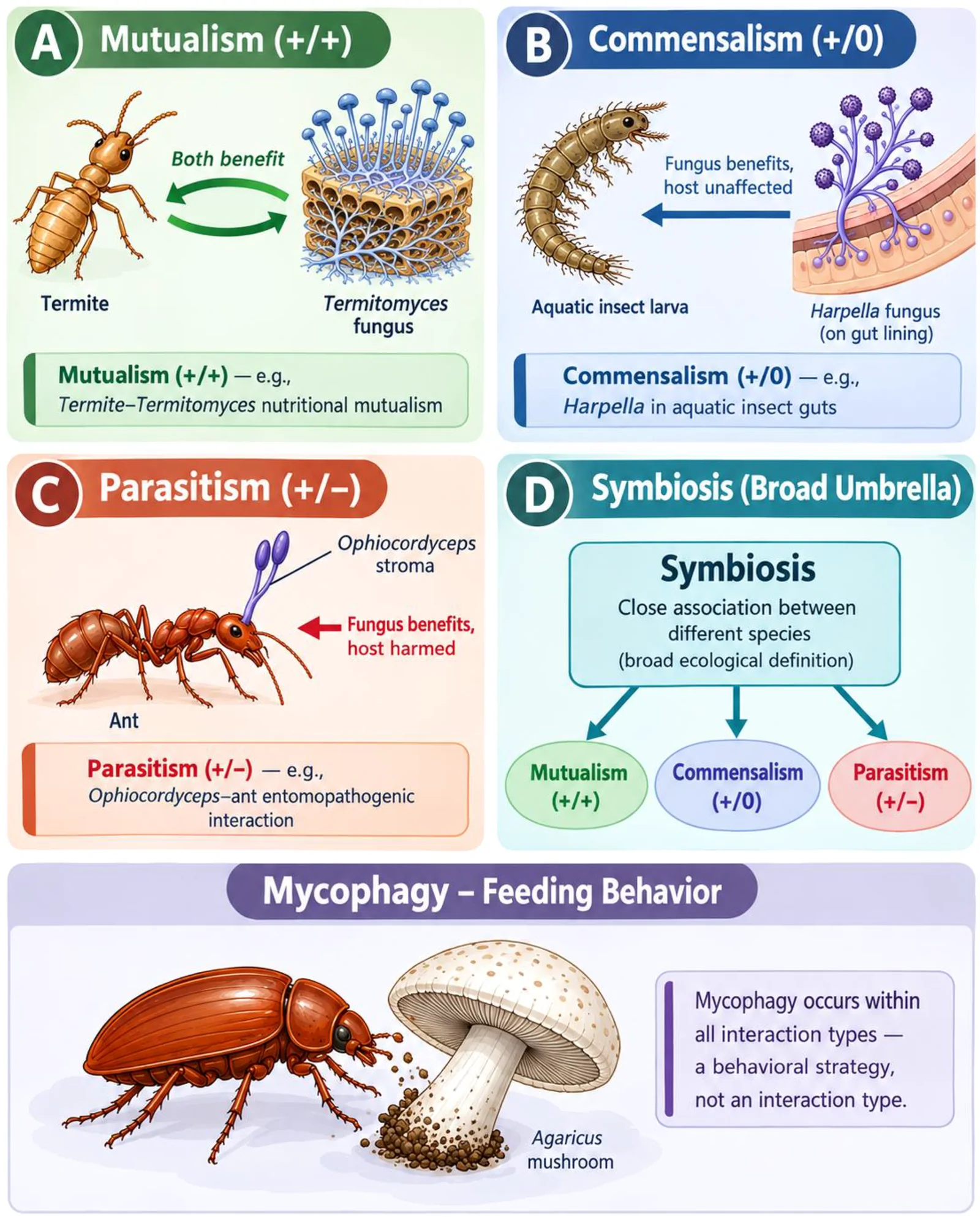
Classification of insect–fungus interactions based on fitness outcomes. **(A)** Mutualism (+/+): both partners benefit (e.g., termite–*Termitomyces* nutritional mutualism). **(B)** Commensalism (+/0): one partner benefits, the other is unaffected (e.g., *Harpella* fungi in aquatic insect guts). **(C)** Parasitism (+/-): the fungus benefits at the expense of the host (e.g., Ophiocordyceps–ant entomopathogenic interaction). **(D)** Symbiosis is used as a broad umbrella term encompassing all three categories. Bottom: Mycophagy (fungus-feeding) is a behavioral strategy that can occur within any of these interaction types, rather than a distinct interaction category. (Created with BioRender.com).

### 4.1 Mutualistic interactions: an overview

Mutualism is an ecological interaction that confers reciprocal fitness benefits to both interacting partners (Biedermann and Vega, 2020).

Within insect–fungal systems, mutualistic associations have evolved at least 14 times independently across Ascomycota and Basidiomycota. These 14 independent origins refer specifically to the evolution of insect farming systems (agricultural mutualisms), such as those observed in ambrosia beetles (Coleoptera), fungus-farming termites (Blattodea), and leaf-cutter ants (Hymenoptera). Among documented mutualistic records, Coleoptera are the most frequent insect participants (27.6%), followed by Hymenoptera (24.1%) and Blattodea (13.8%). Beyond these farming systems, our broader literature synthesis identified 21 independent phylogenetic origins of insect associations within Ascomycota and 12 within Basidiomycota, spanning mutualistic, commensal, and parasitic interactions across six insect orders. The distinction is important: the 14 origins represent the evolution of obligate agricultural symbioses, while the 21 and 12 origins represent all insect-associated lineages within each phylum, including non-farming associations such as gut commensals and entomopathogens.

A unifying framework for the convergent evolution of these associations lies in the shared nutritional challenges posed by insect hosts and their substrates. Across disparate fungal lineages, repeated selection pressures have favored expansions in carbohydrate-active enzyme (CAZyme) families, particularly AA4 (lignin-modifying enzymes), CE4 (acetyl xylan esterases involved in hemicellulose deacetylation), and GH3 (β-glucosidases for cellulose degradation). These enzymatic expansions are consistently observed in insect-associated fungi, regardless of whether they are mutualists (e.g., *Termitomyces* and *Ambrosiella*) or pathogens (e.g., *Metarhizium*, *Beauveria*). This pattern of convergent evolution reflects the universal requirement to degrade lignocellulose-rich plant material (in mutualists) or to breach insect cuticles composed of chitin and proteins (in pathogens). The repeated selection for these CAZyme families across phylogenetically distant fungal classes, Sordariomycetes, Agaricomycetes, and others, demonstrates that ecological convergence, driven by similar nutritional and ecological niches, is a major force shaping insect–fungus associations.

The origin of fungal-insect mutualism remains a mystery, but it is an ancient phenomenon. The association between Ophiostomatales fungi and Platypodinae wood-boring weevils/ambrosia beetles provides the oldest molecular evidence for insect-fungi mutualism (Biedermann and Vega, 2020; Huang et al., 2025). These interactions involve the exchange of services between fungi and insects and are broadly classified into nutrition, protection, and dispersal. Recent phylogenomic analyses have revealed that ambrosia fungi evolved through lineage-specific expansions in carbohydrate-active enzyme families, enhancing their capacity for cellulose degradation and lignin modification in beetle galleries (Huang et al., 2025). Since fungi are inherently sessile, they often form mutualistic associations based on dispersal (Biedermann and Vega, 2020; Koyuncu et al., 2025). An analysis of extant fungal-insect mutualisms has revealed that almost all fungal species in these mutualisms are dispersed by their insect partners, with very few exceptions.

### 4.2 Dispersal mutualisms

One well-characterized example in this regard is the mutualism between several members of the genus *Amylostereum*, such as *A. areolatum*, *A. chailetii*, and *A. laevigatum* and siricid wood wasps such as *Sirex noctilio* primarily for their successful dispersal (Marčiulynas et al., 2024; Wang et al., 2024). During oviposition, the female wasp inoculates the host tree with fungal spores, which in return colonize and degrade woody tissues, thus providing the optimal environment with essential nutrients and enzymes crucial for wasp larvae development and survival (Slippers et al., 2003; Marčiulynas et al., 2024). In return, the fungi benefit from effective dispersal of their propagules and access to new substrates (Slippers et al., 2003; Wang et al., 2024). This interaction is further facilitated by the fungi, which produce detoxifying enzymes, including cytochrome P450S, that mitigate host tree chemical defenses, enabling colonization of the wood (Xie and Lu, 2025). However, the burrowing activity of the larvae and wood degradation by fungi cause significant ecological and economic damage to the host pine trees (Krivak-Tetley et al., 2021; Wilcken et al., 2025). Insect-fungal mutualism can also evolve around reproductive services. This is well illustrated in *Epichloë*, which are heterothallic endophytes that live in grasses and are capable of producing stromata on the grass culms (Miura et al., 2024; Hewitt, 2025; Luna-Fontalvo et al., 2025).

To achieve cross-fertilization, the fungi need to transfer spermatia between individuals from different grass clumps, as only cross-fertilization allows them to produce perithecia and facilitate efficient dispersal and propagation. *Botanophila* flies are attracted to these fungal spermatia for feeding and oviposition (Bultman and Leuchtmann, 2008; Stawrakakis et al., 2023). Upon laying eggs, they exhibit specialized abdominal movements to defecate the spermatia they consume from a different fungal clump onto the stromata, thereby facilitating cross-fertilization of the fungi (Bultman et al., 1998, 2022). This new kind of mutualism between fungi and insects reveals an interaction analogous to angiosperm-insect pollination systems, in which the fly benefits from a food source while the fungus achieves cross-fertilization through insect-mediated sperm transfer (Bultman et al., 1998). However, mutualism comes with costs; in the *Epichloë*-*Botanophila* interaction, larval feeding on fungal perithecia reduces fungal reproductive output, demonstrating that mutualistic outcomes depend on the balance of costs and benefits (Bultman et al., 2000).

Although larval feeding imposes some cost on fungal reproductive success (Stawrakakis et al., 2023), the net interaction remains mutualistic, as it enhances fungal reproductive success and provides nutritional benefits to the insect (Newman et al., 2021). Fungal sex in the *Epichloë-Botanophila* mutualism involves odor signals that attract flies, representing a specialized pollination-like interaction (Hossaert-McKey et al., 2008). Mycophagy by a millipede can impact insect-fungus mutualisms by feeding on fungal reproductive tissues, representing an exploitative interaction that reduces fungal fitness (Bultman and Mathews, 1996). Additional dispersal mutualisms include *Claviceps purpureum*, which attracts insects for spore dispersal via sugary exudate (Lemon, 1992), and *Phallus* spp. attracting flies for spore dispersal via odor (Tuno, 1998), *Puccinia monoica* creating pseudoflowers to attract pollinators (Roy, 1996), and *Agaricus* spp. whose spores are dispersed via insect feces after larvae feed on the fungi (Bunyard, 2007). *Metschnikowia*, *Cryptococcus*, and *Aureobasidium pullulans* influence nectar scent and improve bee fitness while being dispersed by bees (Jacquemyn et al., 2021).

### 4.3 Nutritional mutualisms: agriculture and farming systems

Nutritional mutualisms are the most prevalent type of insect-fungus cooperation, in which fungi provide essential nutrients, detoxify plant allelochemicals, or degrade recalcitrant substrates such as cellulose and lignin. In return, insects provide substrate, habitat, and often vertical transmission.

#### 4.3.1 Ambrosia beetles and bark beetles

One of the most innovative beetle-fungus mutualisms is the development of mycangia, which are specialized structures for storage, transport, and maintenance of fungal symbionts. Mycangia are specialized glands or pouches found in Scolytinae (bark beetles) and Platypodinae (ambrosia beetles) beetles’ heads, thorax, or mandibles, which are used to carry, store, and protect fungal spores or hyphae (Joseph et al., 2025a). The secretions of glandular cells of mycangia nourish and protect fungal counterparts (Joseph et al., 2025b). The adult beetles act as vectors, transporting fungal spores or hyphae as they move between habitats, and are involved in fungal inoculation into a tree host and successful propagation. A recent study of the rare platypodine beetle (*Treptoplatypus oxyurus*) has revealed that its mycangia harbor not only the primary Ophiostomatales symbiont but also multiple yeast species, indicating that these symbiotic communities are more diverse than previously recognized (Kolařík et al., 2026). Experimental validation of Leach’s postulates has confirmed that adult ambrosia beetles naturally carry fungal pathogens on their bodies with both the adults and their progeny capable of inoculating new host plants (Costanzo et al., 2025). Moreover, frass produced by beetles within galleries can store viable fungal propagules for extended periods of time ranging from 122 to over 300 days depending on beetle species, demonstrating that infected trees remain a source of inoculum long after the initial attack (Sofaer et al., 2025; Peck et al., 2026). These fungi can grow well in these galleries feeding on the plant materials by degrading complex polysaccharides such as cellulose and lignin into simple sugars, proteins and other assimilable nutrients thus enabling the beetles to exploit nutrient-poor substrates such as wood. Research studies have demonstrated that beetle-associated fungi possess high enzymatic activities, including endoglucanase (3.39 IU/gds) and xylanase (356.24 IU/gds), enabling efficient saccharification of lignocellulosic substrates into fermentable sugars (Wagner et al., 2025). Genomic analyses in selected fungi support these findings, identifying lineage-specific expansions in CAZyme families (AA4, CE4, and GH3) that enhance lignin modification, hemicellulose deacetylation, and cellulose degradation across different ambrosia lineages (Huang et al., 2025). Patterns of functional enzyme activity in fungus-farming ambrosia beetles reveal that enzymatic profiles vary significantly across gallery compartments and with age, reflecting the dynamic nutritional demands of developing beetle larvae (De Fine Licht and Biedermann, 2012). Furthermore, chemical analysis of the fungal symbiont *Alloascoidea hylecoeti* shows that it is rich in soluble sugars, free amino acids, fatty acids, and B vitamins, compounds that directly nourish beetle larvae developing in nutrient-poor wood substrates (Lehenberger et al., 2025).

Beyond the nutritional mutualism, the ambrosia beetle gallery is a highly dynamic ecological niche shaped by interactions between beetle-vectored fungi, host tree and resident wood microbiome. Studies have shown that the endophytic community can significantly influence the performance of introduced fungal symbionts. A large-scale study encompassing 319 infested trees, 1, 148 *in vitro* assays revealed that wood-inhabiting endophytes including 15 fungal and 11 bacterial species exhibited antagonistic activity against *Fusarium* pathogens vectored by ambrosia beetles (Lynch et al., 2025). Moreover, beetle larvae feed directly on fungal colonies, and experimental data have revealed that specific fungal symbionts, particularly *Fusarium* sp. FL-1 and AF-8, support the highest rates of larval development compared to other associates (Harrington et al., 2021; Cruz et al., 2025). Ambrosia-associated genera such as *Ambrosiella*, *Raffaelea* and *Geosmithia* represent a polyphyletic group demonstrating the diversity of fungal partners that have co-evolved with wood-boring beetles (Joseph and Keyhani, 2021; Huang et al., 2025). New fungus-insect symbiosis discovered in *Ambrosiodmus ambrosia* beetles using culturing, molecular, and histological methods reveals that saprophytic Polyporales fungi serve as mutualists, expanding our understanding of ambrosia beetle-fungus associations (You et al., 2015). Ambrosia fungi play a significant role in the insect-fungi symbiosis related to cork oak decline, where the ambrosia beetle Platypus cylindrus may transport pathogenic fungi to susceptible trees (Henriques et al., 2006). Harrington et al. (2021) showed that several Agaricomycete fungal species (*Entomocorticium* spp., *Cylindrobasidium ipidophilum, Phlebiopsis gigantea*), which are associated with the beetles have evolved non-forcible spore discharge methods, that are absent in wild free-living members of the same genera. Furthermore, some of these mutualistic relationships can be economically important. The relationship between *Ophiostoma ulmi* and the elm bark beetle (*Scolytus* spp.) caused the devastating Dutch Elm Disease, which led to the loss of the American elm as the premier street tree in North America and Europe (Satini and Faccoli, 2015; Pepori et al., 2025; Cambronero-Heinrichs et al., 2025). Effects of mass inoculation on induced oleoresin response in intensively managed loblolly pine reveal that abiotic factors such as nutrient supply and water relations have the potential to modify plant-insect-fungus interactions, influencing tree defense responses against bark beetles and their associated fungi (Klepzig et al., 2005). Thousand cankers disease in walnut trees in Europe, caused by the insect/fungus complex of the walnut twig beetle (*Pityophthorus juglandis*) and the fungal pathogen *Geosmithia morbida*, represents an emerging threat requiring integrated management strategies (Bracalini et al., 2023). **Figure 5** contrasts the structural basis of these farming systems, illustrating the mycangium of ambrosia beetles (Panel A) and the fungus comb of termites (Panel B) and highlighting convergent evolution of specialized morphological adaptations for fungal cultivation.

**Figure 5:**
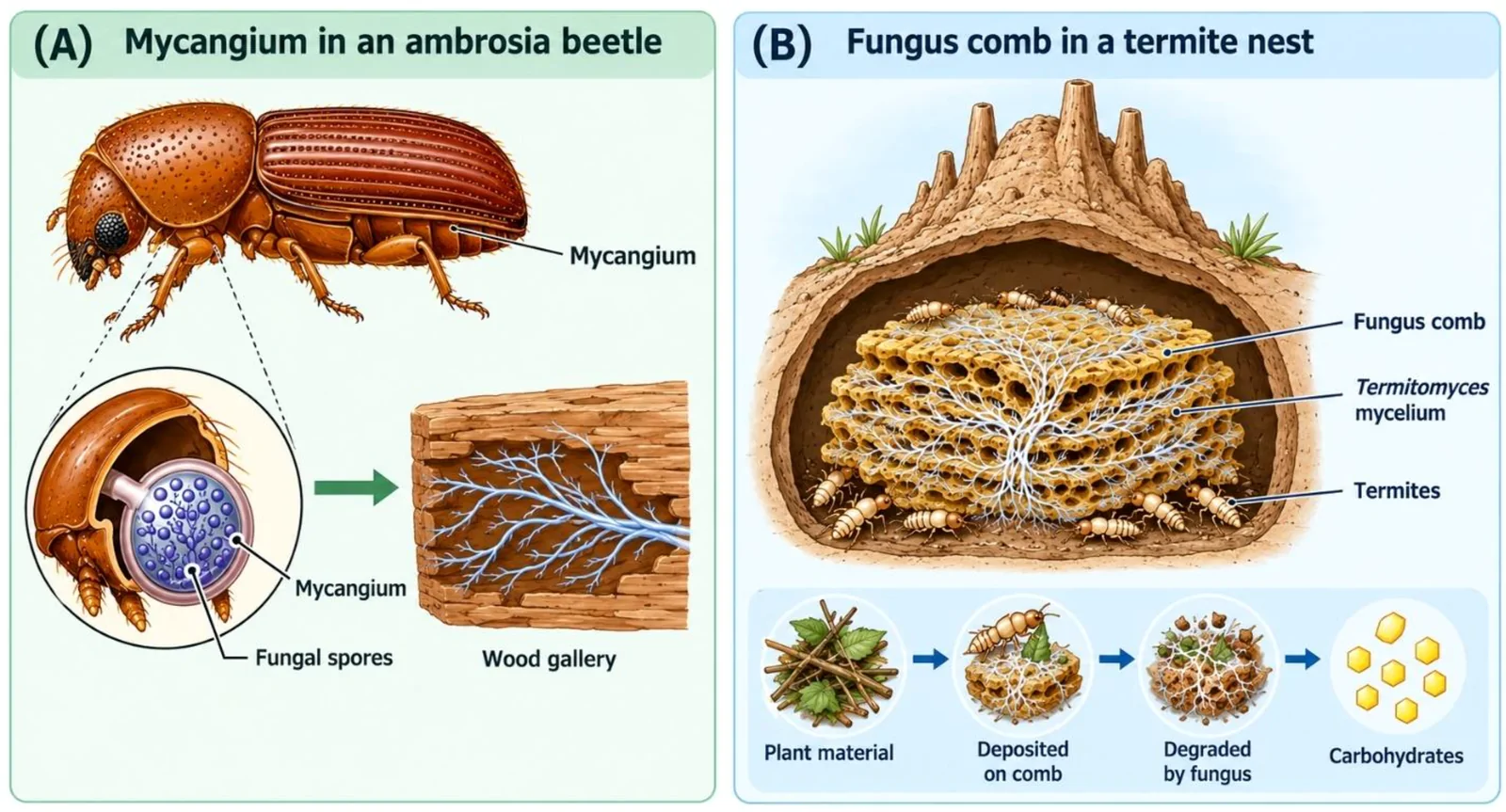
Specialized morphological adaptations in insect–fungus mutualisms. **(A)** Mycangium (spore-carrying organ) in an ambrosia beetle, showing fungal spore storage and inoculation of wood galleries. **(B)** Fungus comb in a termite nest, showing *Termitomyces* cultivation and lignocellulose degradation into digestible carbohydrates. Both systems represent convergent evolution of nutritional mutualisms enabling utilization of nutrient-poor plant substrates (Created with BioRender).

#### 4.3.2 Fungus-farming termites

The mutualistic association between Macrotermitinae termites and *Termitomyces* fungi, involving 14 known fungal species, represents a derived farming system characterized by vertical transmission, enzyme-mediated lignocellulose degradation, and the suppression of defensive symbiont activity (Aanen and Eggleton, 2005; Wood and Thomas, 1989). The ecology of fungus farming by termites encompasses the fungal population genetics and the defensive mechanisms termites use against the parasitic fungus *Pseudoxylaria*, revealing how termites maintain monoculture of their mutualistic fungus while suppressing antagonistic fungi (Katariya, 2018). Observations of termite-fungus interactions are of potential significance for wood biodeterioration and protection, as understanding these associations can inform strategies to prevent termite-fungal complex-caused wood damage (Wong and Check, 2001). Several studies have documented associations between *Trichoderma* and termites (Subekti et al., 2024; Yang et al., 2025; Ribeiro et al., 2025).

The termites cultivate the fungus *Termitomyces* in “fungus combs, “ specialized structures within their nests that provide protection and optimal conditions for growth, dispersal, and vertical transmission (Aanen and Eggleton, 2005; Nobre and Aanen, 2012). Termites cannot completely digest dead plant materials, so they deposit partially degraded plant material onto the fungus comb, providing substrate for fungal growth. The fungus degrades complex lignocellulosic materials that termites cannot break down on their own, converting them into digestible carbohydrates for termite consumption. Termites facilitate fungal dispersal by spreading spores and propagules through their feces and by transporting pieces of the fungus comb during colony expansion (Aanen and Eggleton, 2005; Nobre and Aanen, 2012). Termites are essential for the propagation of *Termitomyces*, as the fungus rarely grows independently without termite involvement (Nobre and Aanen, 2012).

Genetic diversity of termite-egg-mimicking fungi, known as “termite balls, “ within termite nests represents a specialized form of insect-fungus interaction in which fungi produce sclerotia that mimic termite eggs, exploiting host care behavior (Yashiro et al., 2011). The cuckoo fungus mimics termite eggs by producing the cellulose-digesting enzyme β-glucosidase, representing an example of chemical mimicry resulting from insect-fungus association (Matsuura et al., 2009). The cuckoo fungus ‘termite ball’ produces sclerotia that exploit termite social behavior, enabling worker termites to care for the eggs and thereby ensuring fungal dispersal and survival (Matsuura and Yashiro, 2019).

#### 4.3.3 Leaf-cutter ants

A comparable yet independent system has evolved among the leaf-cutter ants, which cultivate *Leucocoprinus* spp. as their primary food source. These ants maintain a fungal colony by providing plant materials, suppressing microbial competitors, and enhancing substrate decomposition with enzyme-rich fecal material. The fungi, on the other hand, produce specialized nutrient-rich structures called gongylidia that sustain the ant colony (Currie et al., 1999). A newly emerging hypothesis on the origin of attine ant–fungus mutualism suggests that the relationship evolved through exploitation of a preexisting insect–fungus symbiosis rather than arising *de novo* (Sánchez-Peña, 2005). Fungus-farming insects have multiple evolutionary origins and diverse evolutionary histories, with independent transitions to agriculture evolving in beetles, termites, and ants (Mueller and Gerardo, 2002). The evolution of agriculture in insects, including fungus-farming ants, termites, and ambrosia beetles, represents a major evolutionary transition where complex coevolutionary interactions between insects and their fungal cultivars have shaped the success of these farming systems (Mueller et al., 2005). The complexity of insect–fungal associations in the attine ant–fungus symbiosis is influenced by the roles of additional microorganisms, including specialized pathogens and commensals (Poulsen and Currie, 2006). Collaborative research on the evolution of adaptive synergism between mutualistic partners during range-limit evolution focuses on understanding how insect-fungus mutualisms respond to environmental gradients and geographic range boundaries (Kellner, 2014).

#### 4.3.4 Gut endosymbionts and yeasts

Another example of a tightly co-evolved mutualistic relationship is observed in *Symbiotaphrina kochii-* and the Cigarette beetle (*Lasioderma serricorne*) and involves strictly vertical transmission of the fungus throughout the beetle’s life cycle, which means the beetle passes the fungus to their eggs, and the larvae get infected with the endosymbiont, which persists throughout the insect’s life cycle (Alina et al., 2026). Residing within the beetle’s gut, the fungus synthesizes essential vitamins and also contributes to the degradation of complex lignocellulosic materials, thereby enhancing the host’s nutrition. In return, the fungus benefits from a stable and protected habitat that supports its growth and transmission (Alina et al., 2026). Symbiosis between yeasts and insects is widespread, with yeasts providing essential nutrients and detoxification services in exchange for insect dispersal (Gonzalez, 2014). The insect-fungus symbiosis between the small hive beetle (*Aethina tumida*) and the yeast *Kodamaea ohmeri* represents an important association in beekeeping, where yeast metabolites may influence beetle behavior and reproduction (Amos et al., 2019). Insect symbiosis involves the derivation of yeast-like endosymbionts within entomopathogenic filamentous lineages, representing a transition from pathogen to mutualist (Suh et al., 2001). *Yarrowia* spp. preserve carcasses and enable larval feeding in burying beetles (Shukla et al., 2018). *Cryptococcus*, *Rhodotorula*, *Debaryomyces, Trametes*, and *Penicillium* spp. in Hemiptera are involved in nitrogen recycling (Vera-Ponce de León et al., 2016). *Cladosporium* spp. improve digestion of cellulose and xylan in *Trichoptera* (Souza et al., 2022) and assist in sugar assimilation in nectar diets of Diptera (Guégan et al., 2020).

The relationship between the associated fungal microbiota and the coffee berry borer beetle (*Hypothenemus hampei*) is a mutualism, in which both parties depend on each other to meet their nutritional requirements. The larval stages of this insect rely on the colonies of *Fusarium solani* for their primary nutrients, which are essential for their growth and development. In return, other members of the microbiota (*F. oxysporum, F. heterosporum, P. echinulatum*, and *F. solani*) benefit from the insect’s burrowing activity in coffee berries, enabling their successful colonization and direct access to their food source (Carrión and Bonet, 2004). Insect-associated fungal symbionts thus represent a promising source of detoxifying enzymes that can degrade plant defensive toxins, insecticides, and other xenobiotics, with potential applications in bioremediation and pest management (Dowd, 1992). Iron acquisition in the weevil-farming fungus *Penicillium herquei* reveals that mineral elements play a previously unrecognized role in insect-fungus mutualistic associations (Qiu et al., 2025). Recent advances in next-generation sequencing and bioinformatics have greatly enhanced our understanding of the genetic factors underlying fungus-insect associations. Comparative analysis of insect-symbiotic fungal genomes has revealed a core gene toolbox comprising genes, including expanded families of carbohydrate-active enzymes, small secreted proteins, and pathogen-host interaction genes, that are exclusive to fungus-insect associations but absent from their closely related free-living members (Wang et al., 2018).

### 4.4 Protective mutualisms

Beyond nutrition, fungal symbionts in some instances offer protective benefits to insects. A good example involves dinidorid stinkbugs, which possess specialized external symbiotic organs on their hind legs that harbor fungi (primarily Cordycipitaceae). Females transfer these fungi to their eggs during oviposition, where the fungi form a barrier against parasitoid wasps (Nishino et al., 2025). Similarly, *Septobasidium* sp. forms mycelial mats on tree bark and twigs, covering scale insects and thereby protecting them from predators and environmental stressors (Murrin, 1996). Some cases of insect-fungus interaction involve some aspect of parasitism that results in balanced association at the population level. For example, *Septobasidium* spp. form extensive mycelial cover over scale insect colonies providing protection against natural enemies and environmental stress while simultaneously parasitizing a subset of individuals to acquire nutrients. Such a balance of costs and benefits maintains a balanced population on both sides and constitutes a form of conditional mutualism (Murrin, 1996). Obligatory mutualisms are another variant of mutualistic association in insect-fungus and are observed between *Botryosphaeria dothidea* and gall midge (*Asteromyia carbonifera*). In this system, fungal infection is a requirement for gall formation, while insect larvae depend on fungal tissues for nutrition. The female midges collect fungal conidia, store them in mycangia on their ovipositors, and release fungal spores to new host plants during oviposition. Upon hatching, the larvae rely on the fungus for nutrition, and the gall it produces serves as a protective structure against environmental conditions and natural enemies such as parasitoids (Heath and Stireman, 2010). Fungal phytopathogens can modulate both plant and insect responses to promote their own dissemination, establishing a new paradigm for plant-insect-fungus associations (Franco et al., 2021). Pathogen-induced pseudoflowers and buttercups can influence each other’s insect visitation patterns, with both plant species receiving greater visitation when associated than when alone, demonstrating complex facilitative interactions among plants, fungi, and insects (Roy, 1994). Modification of coevolved insect-plant interactions by an exotic plant pathogen can alter established ecological relationships, with the fungus indirectly affecting insect behavior and plant susceptibility (Storer et al., 1999). Insect-fungus blister galls on Solidago species represent a specialized host-parasite relationship in which fungal infection induces gall formation that benefits both the fungus and the insect host (1981). The evolutionary ecology of the *Botryosphaeria dothidea* mutualism with the goldenrod-galling midge *Asteromyia carbonifera* represents a highly specialized insect-fungus interaction (Janson, 2010). The laurel wilt disease represents a complex tripartite interaction between the fungal pathogen (*Raffaelea lauricola*), its ambrosia beetle vector, and susceptible host trees, offering insights into the complexities of fungus-insect-plant interactions (Abdrabo et al., 2024).

### 4.5 Commensalism

Commensal organisms are symbionts generally considered to have a neutral impact on their hosts, neither causing harm nor providing measurable benefits (Veiga, 2016). However, recent research has challenged this oversimplification. Emerging experimental and ecological evidence suggests that many of the associations traditionally categorized as commensalism can quite often shift along a continuum toward either mutualism or parasitism depending on environmental conditions, host physiology, and community context, suggesting that commensalism may represent an undetected or context-dependent interaction rather than a truly neutral one (Rooy et al., 2025).

To move beyond anecdotal observations, theoretical models of conditional mutualism provide a framework for understanding these context-dependent shifts. According to the nutrient exchange threshold model, mutualistic outcomes are maintained when the nutritional benefits provided by the symbiont exceed the metabolic costs incurred by the host. When environmental conditions alter this balance, for example, under nutrient limitation or thermal stress, the interaction may shift toward commensalism or even parasitism as the host’s immune system becomes compromised and the fungus exploits weakened defenses. Similarly, the host immune cost model predicts that environmental stressors (e.g., temperature extremes, pesticide exposure, or nutrient scarcity) impose physiological constraints on host immunity, reducing the host’s ability to regulate fungal populations within the gut or tissues. Under such conditions, a normally benign commensal may proliferate and become pathogenic. This framework explains empirical observations such as the shift of *Zancudomyces culisetae* from commensalism to mutualism in nutrient-limited mosquito larvae (Rooy et al., 2025), and the transition of opportunistic fungi like *Aspergillus* spp. from harmless gut inhabitants to pathogens in immunocompromised insects. These theoretical perspectives elevate the study of insect–fungus commensalism from descriptive accounts to a predictive framework, emphasizing that interaction outcomes are dynamic and environmentally contingent.

In insect-fungus systems, the most commonly observed commensalism involves fungi living in association with insects, typically within the insect’s gut without causing overt harm to the host (Mondal et al., 2023). These gut-associated fungi are thought to obtain nutrients from ingested material or host-derived substrates, with no detectable impact on host fitness.

Fungal genera such as *Harpella* and *Stachylina* (class Harpellomycetes), have been shown to engage in commensal interactions with a wide range of aquatic insects, especially those belonging to the orders Diptera, Ephemeroptera, Plecoptera, and Coleoptera. The fate of fungal spores in the insect gut varies considerably depending on fungal species and host physiology, with some spores germinating and establishing commensal relationships while others are digested or pass through unharmed (Dillon and Charnley, 1991). Recent studies have demonstrated that fungal secondary metabolites can also modulate insect endosymbiont communities. Jesu et al. (2025) showed that *Trichoderma* metabolites 6-pentyl-α-pyrone and harzianic acid significantly reduce the titer of “*Candidatus Erwinia dacicola*” in the olive fruit fly *Bactrocera oleae*, affecting both insect fitness and subsequent generation development. This suggests that fungal metabolites can be formulated for direct or indirect control strategies targeting both pests and their symbiotic partners. The CIGAF database provides a comprehensive platform and interactive resource for insect-associated trichomycete fungi, enabling large-scale analyses of insect-fungus symbiosis, biogeography, and ecological niche differentiation (Chaudhary et al., 2023). These fungi mostly colonize the hindgut of larval hosts and persist as non-invasive symbionts, receiving nutrients from host ingesta with no apparent impact on the host’s survival and fitness (Zhu and Xu, 2025). Commensal gut fungi play an indispensable role in mediating tripartite interactions among plants, fungi, and herbivorous insects, often influencing digestion, detoxification, and host immunity. The insect gut-dwelling fungus *Zancudomyces culisetae* plays a hidden role in mosquito development, influencing larval growth and survival (Rooy et al., 2025). While this association between *Z. culisetae* has traditionally been regarded as commensalism, recent studies provide evidence of a shift from commensalism to mutualism. A study involving *Aedes aegypti* and *Z. culisetae* revealed that mosquito larvae colonized by the fungus exhibited larger body sizes and accelerated development compared to the controls. These findings were consistent under both nutrient-rich and nutrient-deficient conditions (Rooy et al., 2025). Fungal genera, *Austrosmittium*, and *Smittium* (Order Harpellales), are found in the guts of immature aquatic insects such as mosquitoes (Culicidae), blackflies (Simuliidae), and chironomids (non-biting midges). These fungi exhibit comparable life-history strategies, attaching to the hindgut lining and absorbing nutrients from the gut contents without penetrating host tissues. They then complete their life cycle when the host molts and new spores are dispersed into the water (Wang et al., 2019).

### 4.6 Entomopathogenic fungi (parasitism)

Insects are susceptible to infection by a wide range of obligate or facultative entomopathogenic fungi, which exploit insect hosts as their primary or exclusive nutrient source (Nicoletti and Becchimanzi, 2022). Entomopathogenic fungi do not constitute a monophyletic clade but rather are phylogenetically diverse and are distributed across multiple phyla, including Chytridiomycota (65 species), Microsporidia (339 species), Entomophthoromycota (474 species), Basidiomycota (238 species), and Ascomycota (476 species) (Araújo and Hughes, 2016) (Oomycetes, although historically studied alongside entomopathogenic fungi, are not true fungi and are therefore excluded from this list). The most common entomopathogenic fungal species within the phylum Ascomycota are *Metarhizium* (*M. anisopliae, M. robertsii, M. brunneum, M. lepidiotae, M. globosum, M. acridum, M. majus, M. flavoviride, M. rileyi, M. pingshaense, M. lepidiotae* and *M. guizhouense*), *Beauveria (B. bassiana* and *B. brongniartii), Isaria (I. fumosorosea*—formerly *Paecilomyces fumosoroseus*, *I. farinosa* and *I. tennuipes), Ophiocordyceps (O. sinensis*—formerly *Cordyceps sinensis*, and *O. unilateralis*), *Cordyceps* (*C. militaris*), *Torubiella (T. ratticaudata), Pochonia (P. chlamydosporia)*, *Lecanicillium (L. lecani*, formerly *Verticillium lecanii, L. longisporum)*, *Hirsutella (H. thompsonii, H. nodulosa*, and *H. aphidis)*, and *Paecilomyces* (*P. variotii*, and *P. lilacinum*) (Khan et al., 2012; Tkaczuk et al., 2015; Jaihan et al., 2016).

The major ecological role of entomopathogenic fungi is their biological control of different insect populations (Litwin et al., 2020). Entomopathogenic fungi serve as major mortality agents in insect populations, and understanding the ecological determinants of these interactions should be prioritized for effective biological control (Rizal et al., 2022; Gielen et al., 2024). A comprehensive review of entomopathogenic fungi highlights their diverse interactions with insects and wide-ranging applications in biological control, from agricultural pests to disease vectors (Mantzoukas et al., 2022). Scanning electron microscopy (SEM) studies of the bioagents *Aspergillus niger* and *Penicillium oxalicum* against the Mediterranean fruit fly (*Ceratitis capitata*) adults reveal detailed mechanisms of fungal spore adhesion, germination, and cuticle penetration at the ultrastructural level (El-Akhdar and Ouda, 2008). The infection process in entomopathogenic fungi is complex and involves multiple routes of entry. Entry via enzymatic degradation of the insect cuticle is the primary and best-characterized route of infection. However, alternative routes of transmission exist, including ingestion via the gut and transovarial transmission (Mannino et al., 2019). Fungal infection dynamics are highly temperature-dependent, affecting spore germination, growth, and virulence. Using the lepidopteran model *Galleria mellonella*, Kryukov et al. (2018) showed that entomopathogenic fungi such as *Beauveria bassiana* and *Metarhizium robertsii* exhibited optimal activity at 25 °C, with significantly reduced efficacy at 15 °C and 30 °C. This temperature sensitivity has important implications for biological control, as field efficacy depends on matching fungal strains to local climatic conditions. Interactions between entomopathogenic fungi and arthropod natural enemies can be antagonistic (fungi infecting predators/parasitoids) or synergistic (fungi weakening pests, making them more vulnerable to predation), with significant implications for their combined use in integrated pest management (Furlong and Pell, 2005; Rizal et al., 2022). Microbial signaling plays a crucial role in tri-trophic interactions among insects, fungi, and plants, enabling chemical communication that can be harnessed for pest control (Zhang et al., 2026b). Interactions between coccinellids (ladybirds) and insect-parasitic fungi can be antagonistic, as entomopathogenic fungi may infect ladybirds as non-target hosts, potentially reducing predator populations and undermining biological control programs (Roy and Cottrell, 2008). The coevolution of fungal entomopathogens and their hosts has led to the evolution of a wide range of complex strategies to overcome insect immune responses and behavioral defenses (Vilcinskas, 2019). The pathogenesis of entomopathogenic fungi is complex and involves multiple infectious agents, with adhesins, lytic enzymes, and secondary metabolites playing key roles (Litwin et al., 2020). Proteolytic enzymes and toxins produced by fungi during infection disrupt the host immune system by modulating the cellular and humoral immune responses of the host insect (Shang et al., 2015; Boguś et al., 2017). Some opportunistic pathogens, such as *Aspergillus* spp. (Eurotiomycetes, Aspergillaceae) have likewise been reported to have a regulatory impact on honeybee immune systems, interfering with the insects’ melanization response (Shelby and Popham, 2006; DellaGreca et al., 2019). Some entomopathogenic fungi produce insecticidal toxins. For example, *Beauveria bassiana* produces oosporein, a dibenzoquinone toxin that suppresses mosquito midgut immune responses. This suppression allows gut bacteria to translocate into the hemocoel, leading to dysbiosis and subsequent septicemia (Nicoletti and Becchimanzi, 2022).

The genus *Cordyceps*, represents a particularly important group of entomopathogenic fungi due to its known ecological and medicinal significance. Beyond *Cordyceps*, the genus *Trichoderma* has emerged as a multipurpose microorganism with significant potential for sustainable agriculture (Woo et al., 2023). While traditionally recognized for its mycoparasitic and plant growth-promoting activities, *Trichoderma* species also exhibit insecticidal properties through the production of secondary metabolites that can directly affect insect pests or indirectly enhance plant defense responses. These multifaceted interactions position *Trichoderma* as a valuable component of integrated pest management strategies.

These fungi are grown artificially not only as a source of food but also for their perceived medicinal and health benefits, or as dietary supplements. In the artificial cultivation systems, substrates such as cereal grains are typically employed due to their availability and ease of handling. Nevertheless, supplementation with insect-derived materials (e.g., pupae) has been investigated as a strategy to more closely replicate natural nutritional conditions and to enhance the biosynthesis of key bioactive metabolites, including cordycepin. This is because these fungi naturally parasitize insects, utilizing host tissues as their primary source of nourishment (Jian and Li, 2017). A dedicated review on insect-fungus interactions with special emphasis on Cordyceps spp. highlights the medicinal and economic importance of these entomopathogenic fungi (Kumar et al., 2016). The occurrence and diversity of insect-associated fungi in natural soils in China reveal a rich reservoir of entomopathogenic and symbiotic species (Sun and Liu, 2008).

### 4.7 Other antagonistic interactions (non-lethal)

Approaching risk assessment of complex disease development in horse chestnut trees requires a chemical ecologist’s perspective, as new interactions between insects and phytopathogenic fungi can result in novel forms of complex disease (Johne et al., 2008). Efficient transmission of an introduced pathogen via an ancient insect-fungus association has been documented in cypress cones, where fungus-infected cones harbor seed insects that facilitate pathogen spread (Battisti et al., 1999). Insects act as vectors of plant pathogens in both mutualistic and antagonistic interactions, with tripartite dynamics among insects, fungi, and host plants shaping the disease outcome (Kluth et al., 2002). Leaf functional traits, insect herbivory, and fungal damage on early Eocene leaf compression fossils from Dolus Hill, Wyoming, provide new insights into fungus–insect–plant interactions in the fossil record, demonstrating that these associations have persisted for millions of years (Richbourg et al., 2025). Mutualism between the phytopathogenic fungus *Botrytis cinerea* and the vineyard pest *Lobesia botrana* results in positive effects on insect development and oviposition, demonstrating that fungal infection can benefit insect herbivores by improving host plant quality (Mondy et al., 1998). The *Valsa* canker fungus plays an important role in the growth and development of *Euzophera pyriella* (Lepidoptera: Pyralidae), serving as a mediator in plant-herbivore interactions and demonstrating how fungal pathogens can indirectly benefit insect herbivores while harming the host tree (Hou et al., 2015). Feeding insects with a phytopathogenic fungus can influence their diapause and population dynamics, as demonstrated in the vineyard where the plant-insect-fungus interaction between grapevine, European grapevine moth, and *Botrytis cinerea* affects insect development and survival (Mondy and Corio-Costet, 2004). The role of weevils in the infection process of the fungus *Phomopsis subordinaria* in *Plantago lanceolata* demonstrates how insects can serve as critical vectors, facilitating fungal entry and disease establishment in host plants (de Nooij, 1988). Insect dissemination of fungal pathogens of trees, as exemplified by Dutch elm disease (*Ophiostoma ulmi* transmitted by Scolytidae beetles), represents a classic and economically devastating insect-fungus association (Webber and Gibbs, 1989). Fungi can reduce the preference and performance of insect herbivores on infected or challenged plants, as such plants exhibit altered chemical and physiological profiles that reduce herbivore preference, thereby affecting insect survival and reproduction (Fernandez-Conradi et al., 2018).

Beyond bipartite interactions, symbiotic bacteria often regulate insect–fungus mutualisms, modulating outcomes through chemical signaling and nutrient provisioning. Indeed, emerging evidence suggests that these interaction systems frequently involve additional, often cryptic partners such as bacterial symbionts. Consequently, insect-fungus relationships are more appropriately interpreted within a broader ecological framework that extends beyond pairwise interactions (Chen et al., 2023). The bark beetle mycobiome is among the well-characterized insect–fungus symbioses, for which collaborative research has identified priority areas, including fungal diversity, functional roles, and management implications (Hulcr et al., 2020). Mutualism and antagonism coexist in ecological interactions among bark beetles, mites, and fungi, with each player influencing the others’ fitness and population dynamics (Klepzig et al., 2001). The interaction among ambrosia beetles, host tree species, and their microbiomes represents a complex multi-partite system, where existing knowledge gaps regarding interactions with other microorganisms and host tree physiology remain to be fully elucidated (Cambronero-Heinrichs et al., 2025). Bark beetle-fungus symbioses encompass a diverse array of interactions including antagonism and mutualism, with specialized structures ensuring continuity of the association across generations (Six, 2003). Wood-inhabiting insects can serve as vectors for decomposer fungi, with many wood-inhabiting fungi dispersed primarily by insects rather than wind (Jacobsen et al., 2017). Insect–fungus relationships in wood decomposition involve complex successional patterns, with different species acting as primary decomposers, secondary invaders, and nutrient cyclers (Swift et al., 1979). The evolution of insect-fungus relationships in the primary invasion of forest timber involves specialized colonists that dominate early stages of xylem decomposition in standing trees and freshly cut logs (Dowding, 1984). The structure and outcome of bark beetle–fungus symbioses are shaped by multiple environmental, ecological and evolutionary determinants including host tree chemistry, beetle mycangial morphology, and fungal competitive interactions and abilities (Six, 2012). The role of phytopathogenicity in bark beetle–fungus symbioses challenges the classic paradigm that these fungi are primarily non-pathogenic, with emerging evidence suggesting that some fungal symbionts may actively contribute to tree decline and death (Six and Wingfield, 2011). These interactions are context-dependent, with environmental conditions and host tree status influencing where they fall along a continuum from mutualism to antagonism (Klepzig and Six, 2004). Furthermore, mutual interactions between an invasive bark beetle and its associated fungi affect beetle behavior, reproductive success, population dynamics, and evolution (Wang et al., 2012b). Adaptive diversification in the scolytine-fungus associations has involved changes in feeding ecology, progressive specialization of mycangial structures, and increased dependence on fungal symbionts (Berryman, 1989). The complexity of three-way associations among fungal endophytes, host plants, and insect herbivores is illustrated in **Figure 6**. These interactions involve direct and indirect effects: endophytes can prime plant defenses, alter plant chemistry, and directly affect insect fitness through mycotoxin production, while herbivory can influence endophyte colonization dynamics.

**Figure 6:**
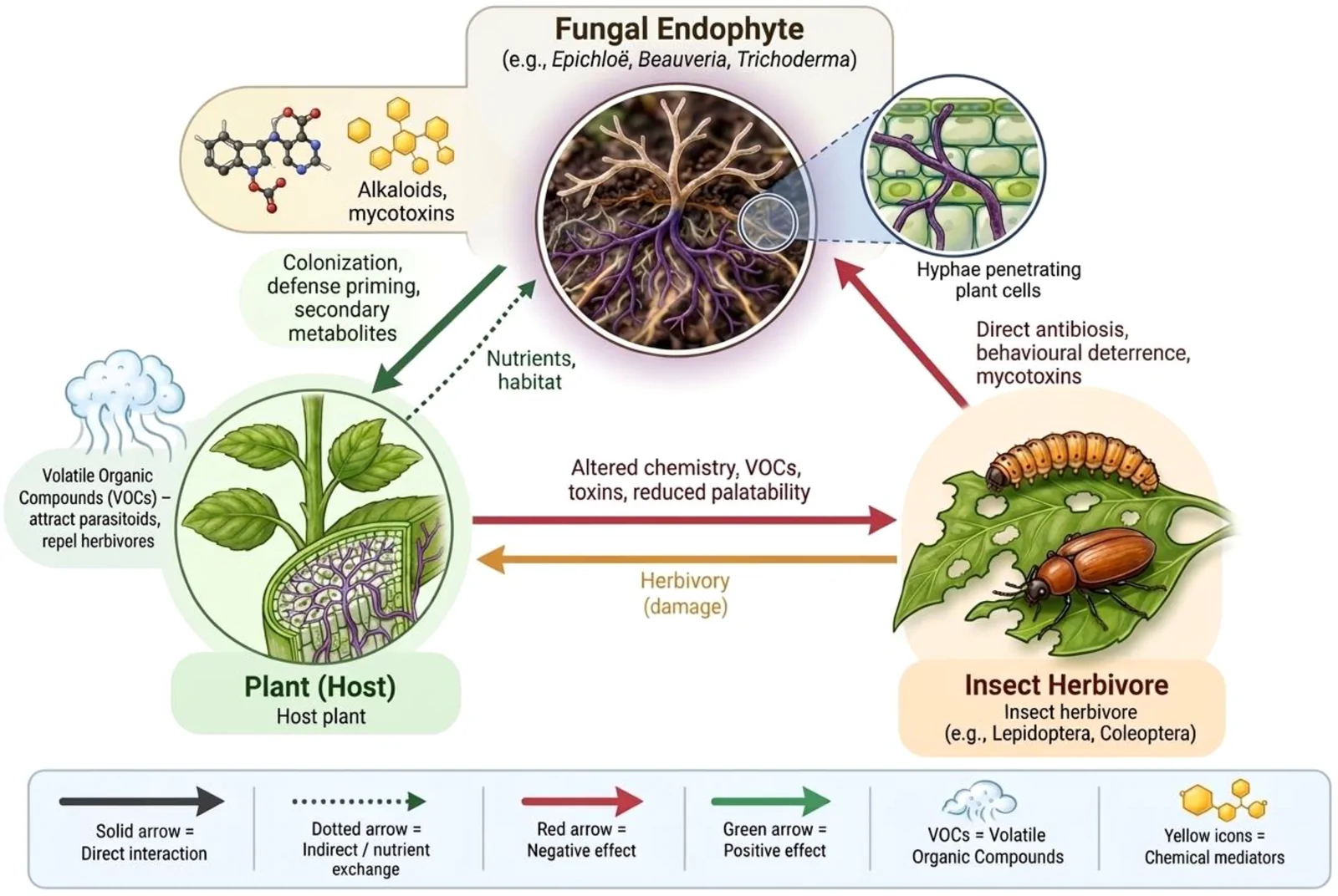
Tripartite interactions among fungal endophytes, plants, and insect herbivores. Endophytes colonize plants (green arrow), alter plant chemistry and volatile profiles (plant → insect, red arrow), and directly affect insects via antibiosis and mycotoxins (endophyte → insect, red arrow). Herbivory (insect → plant, orange arrow) and nutrient exchange (plant → endophyte, dotted green arrow) complete the network. VOCs and secondary metabolites are key chemical mediators (Created with BioRender).

Not all insect–fungal associations that are detrimental to the insect involve classical entomopathogenesis. Some fungi engage in antagonistic interactions that elicit defensive behaviors or reduce insect fitness without necessarily causing rapid mortality. For example, *Trichoderma* metabolites have been shown to act as semiochemicals that elicit aggregation behavior in termites, and termites in turn exhibit burying defenses against *Trichoderma* invasion, suggesting a dynamic antagonistic relationship (Zhang et al., 2023; Yang et al., 2025; Siddika et al., 2025). Similarly, fungal volatile organic compounds (VOCs) can mediate insect oviposition preference, sometimes deterring oviposition on unsuitable substrates, representing another form of non-lethal antagonism (Gu et al., 2022). Beyond direct metabolite effects, Di Lelio et al. (2023) demonstrated that colonization of tomato plants by *Trichoderma afroharzianum* induces dysbiosis in the gut microbiota of *Spodoptera littoralis* larvae, reducing the abundance of *Enterococcus casseliflavus* and thereby disrupting the insect’s nutritional support. This novel mechanism of indirect biocontrol modulates the insect’s microbial community rather than acting through direct toxicity, and represents an emerging strategy for pest management that targets the pest’s symbiotic partners. These examples highlight that antagonistic insect–fungal interactions extend beyond obligate parasitism and include a range of context-dependent, non-lethal negative outcomes.

Insect-fungus symbiosis involving the diversity and potential ecological roles of the hypocrealean fungus Trichoderma in colonies of termites (Blattodea: Termitidae) reveals complex antagonistic and mutualistic interactions that influence colony health and nutrition (Pereira, 2018). *Trichoderma* is a soil-inhabiting and wood-inhabiting fungus known to produce various enzymes (Tyśkiewicz et al., 2022. In plant–fungal interactions, these enzymes facilitate plant resource acquisition and growth stimulation (Tarafder et al., 2024; Gutiérrez-Moreno et al., 2025; Kumari et al., 2025; Sun et al., 2025). In some cases, enzymes produced by *Trichoderma* species can decompose cellulose, which benefits organisms that consume cellulose substrates (Xie et al., 2014; Oliveira et al., 2025). Beyond direct infection, entomopathogenic fungi can also engage in indirect and context-dependent interactions that ultimately harm the insect. For example, fungi can engage in interference competition, in which fungal secondary metabolites play a crucial role in suppressing insect feeding and survival (Trienens and Rohlfs, 2012). Fungal allelochemicals play a critical role in insect pest management, and understanding the chemical ecology of insect-fungus interactions can reveal new targets for mycobiocontrol strategies (Holighaus and Rohlfs, 2016). The perplexing metabolomes in fungal-insect trophic interactions represent a terra incognita of mycobiocontrol mechanisms, and metabolomic frameworks have revealed previously unknown interaction biomarkers (Singh et al., 2016). Density-dependent insect-mold interactions significantly affect fungal growth and spore production, with higher insect densities leading to increased competition and reduced fungal reproductive output (Rohlfs, 2005). Metabolomics reveals insect metabolic responses to fungal infection, providing insights into the biochemical dynamics of host-pathogen interactions (Xu et al., 2015). Induced fungal resistance to insect grazing involves reciprocal fitness consequences and changes in fungal gene expression, as demonstrated in the *Drosophila*-*Aspergillus* model system (Caballero Ortiz et al., 2013). Nutritional changes in response to Drosophila melanogaster feeding on different myxomycetes under environmental stress reveal how fungal food sources can directly or indirectly affect insect-fungus relationships, influencing insect development and survival under stressful and non-stressful conditions (Güneş and Eroğlu, 2026). Fruit, flies, and filamentous fungi, using *Drosophila melanogaster* and *Aspergillus* molds as a model system, provide experimental analysis of animal-microbe competition, helping to disentangle basic principles of antagonistic insect-fungus interactions that have remained unexplored (Trienens et al., 2010). Experimental evolution of defense against a competitive mold in *Drosophila* larvae confers reduced sensitivity to fungal toxins but no increased resistance, revealing that adaptation to fungal competitors can evolve through physiological tolerance rather than direct resistance mechanisms (Trienens and Rohlfs, 2011). Fungal secondary metabolism in the context of animal–fungus interactions serve ecological functions including defense against fungivory, interference competition, and chemical communication (Rohlfs, 2015). Additionally, indirect interactions between insect herbivores and pathogenic fungi on leaves can be either inhibitory or facilitatory, depending on the timing and sequence of attack. For example, prior fungal infection may enhance or reduce subsequent insect herbivory, and vice versa, creating complex dynamics that affect host plant fitness (Hatcher and Ayres, 1997). Multilayered interactions between Lepidoptera and fungi encompass spore dispersal, mycophagy, and entomopathogenic relationships, revealing diverse ecological strategies across this insect order (Koyuncu et al., 2025).

### 4.8 Insect–fungus associations in agriculture and postharvest systems

Beyond natural ecosystems, insect–fungus interactions have significant practical implications for agricultural productivity and postharvest food security. These associations can be either detrimental (causing crop damage and spoilage) or, in some cases, beneficial (offering biocontrol opportunities). Understanding these interactions is critical for developing integrated pest management strategies.

Insect-fungus associations significantly influence seed deterioration, with interactions occurring at preharvest anthesis and during storage, affecting seed quality and germination (Mills, 1983). Postharvest insect-fungus associations affecting seed deterioration involve complex interactions that can significantly reduce seed quality and viability during storage (Mills, 1986). Insect-fungus associations influencing seed deterioration involve complex interactions between storage fungi and insect pests that reduce seed quality and viability (Mukherjee and Nandi, 1993). In agricultural systems, soybean growth, nodule development, and N2-fixing ability can be significantly damaged by an insect-fungus-nematode pest complex, with significant interactive effects among pests (Russin et al., 1990). Insect and mycoflora interactions in stored maize flour involve complex associations between spoilage fungi and stored product insects, with implications for food safety (Allotey et al., 2001). *Aspergillus flavus* plays an unpredictable role in insect-fungus interactions and agroecosystems, and, despite being widespread and economically important, its interactions with insects such as corn earworm and fall armyworm remain poorly understood, highlighting the need to rethink management approaches in complex agroecosystems (Bush, 2022). Combining competition from ryegrass with an insect–fungus combination can effectively control *Rumex obtusifolius* seedlings, with potential synergistic interactions (Keary and Hatcher, 2004). The interactions among insects, fungi, and nematodes contribute to the deterioration of plant roots, with additive rather than simple interactive effects among the organisms (Leach et al., 1963). Collectively, these examples underscore the need for holistic management approaches that consider both insect and fungal components simultaneously, as ignoring either partner can lead to ineffective control and unexpected crop losses.

### 4.9 Resistance evolution in insect-fungal interactions

Insect populations can evolve resistance to entomopathogenic fungi through multiple mechanisms, including cuticle thickening, immune system enhancement, and behavioral avoidance. Experimental evolution studies in *Drosophila melanogaster* have demonstrated reduced sensitivity to fungal toxins following selection pressure, though not increased resistance, suggesting physiological tolerance rather than direct resistance mechanisms (Trienens and Rohlfs, 2011). Field populations of *Plutella xylostella* have shown reduced susceptibility to *Beauveria bassiana* after repeated exposure, with resistance ratios ranging from 2.5- to 8.7-fold (Furlong and Pell, 2005). However, compared to chemical insecticides, where resistance ratios can exceed 1, 000-fold, fungal biocontrol agents exhibit relatively low resistance potential due to their multi-mechanistic infection process involving cuticle degradation, toxin production, and immunosuppression (Vilcinskas, 2019). Resistance management strategies including strain rotation, combination with other biocontrol agents, and integration with cultural practices should be considered for sustainable use (Mantzoukas et al., 2022).

### 4.10 Non-target impacts and environmental safety

While entomopathogenic fungi are generally considered environmentally safe due to their host specificity, non-target effects on beneficial insects including pollinators (bees, bumblebees) and natural enemies (parasitoids, predators) have been documented. *Beauveria bassiana* and *Metarhizium anisopliae* can infect non-target Lepidoptera, Coleoptera, and Hymenoptera under laboratory conditions, though field effects are typically limited (Roy and Cottrell, 2008). Ladybird beetles (Coccinellidae) show variable susceptibility, with some species exhibiting lower infection rates due to cuticular antifungal compounds (Roy and Cottrell, 2008). Parasitoid wasps can be infected when ovipositing into hosts infected with fungi, although horizontal transmission to uninfected hosts is limited (Furlong and Pell, 2005). Risk assessment frameworks based on the IOBC (International Organization for Biological Control) categorization indicate that most entomopathogenic fungi are “harmless” to “slightly harmful” to beneficial arthropods (Hassan et al., 1994). Environmental factors including application timing, formulation, and fungal strain selection are critical for minimizing non-target impacts (Rizal et al., 2022).

### 4.11 Commercialization, registration, and economic feasibility

Commercial development of entomopathogenic fungi faces several challenges:

#### 4.11.1 Registration barriers

Regulatory requirements for fungal biocontrol agents vary significantly across jurisdictions. In the EU, registration under Regulation (EC) 1107/2009 requires extensive ecotoxicological, residue, and efficacy data, with estimated costs exceeding €10 million per product (Sundh and Goettel, 2013). Risk assessment of genetically modified entomopathogenic fungi presents additional regulatory challenges. Ahmad et al. (2025) emphasized that rigorous evaluation of modified biocontrol fungi is essential to prevent harm to beneficial insects, particularly pollinators, and to avoid unintended ecological disruption. The complexity of assessing transgenic fungal strains in natural environments remains a significant hurdle for regulatory approval. In the USA, EPA registration requires mammalian toxicity, non-target organism testing, and environmental fate studies, with costs ranging from $500, 000 to $2 million (U.S. Environmental Protection Agency (EPA), 2021). China’s Ministry of Agriculture and Rural Affairs requires field efficacy trials (≥3 locations), quality control testing, and toxicological assessment, with registration taking 3–5 years (Tan et al., 2022).

#### 4.11.2 Commercialization challenges

Production costs remain a significant barrier. Solid-state fermentation yields 10³-10^4^ conidia/g substrate, while liquid fermentation yields 10^8^-10^9^ conidia/mL but requires careful optimization of carbon/nitrogen ratios, oxygen transfer, and harvesting (Mascarin and Jaronski, 2016). Shelf-life limitations (6–18 months at 4 °C) reduce commercial viability compared to chemical pesticides (2–5 years) (de Faria and Wraight, 2007).

#### 4.11.3 Economic feasibility

The global market for mycoinsecticides was valued at $1.8 billion in 2023 and projected to reach $3.2 billion by 2030 (CAGR 8.7%) (Mordor Intelligence, 2024). Saminathan et al. (2025) emphasized that despite significant research progress, the translation of entomopathogenic fungi from laboratory to field applications faces challenges including inconsistent field performance, formulation stability under varied environmental conditions, and farmer acceptance. Addressing these constraints requires collaborative efforts between researchers, industry, and regulatory bodies. Field trials of *Beauveria bassiana* against grasshoppers and locusts in Cape Verde demonstrated approximately 45% reductions in pest densities, confirming the efficacy of these biopesticides under field conditions. Similarly, *Metarhizium acridum* has been used operationally in Australia since 2000, with nearly 80, 000 ha treated. However, adoption remains limited by inconsistent field performance and farmer perception of higher risk compared to chemical alternatives (Vega, 2018).

## 5 Discussion

Insects and fungi represent two of the most diverse and ecologically successful lineages in the Tree of Life and their interactions span a remarkable continuum of interaction outcomes (Biedermann and Vega, 2020). Foundational works by Wood and Thomas (1989) and Cherrett et al. (1989) established the framework for understanding the diversity and complexity of insect-fungus associations and their functional significance. The comprehensive volume “Insect-Fungus Interaction” edited by Beaver and colleagues further consolidated knowledge of the ecological and evolutionary aspects of these associations, bringing together diverse perspectives from entomology and mycology (Beaver, 1989). Subsequent decades of research in the area have substantially expanded this framework, demonstrating that fungal symbioses are key to multiple dimensions of insect biology (Klepzig et al., 2009; Biedermann and Vega, 2020). Symbiosis catalyses niche expansion and diversification, with insect-fungus associations serving as a prime example of how intimate interactions can enable exploitation of novel resources and drive evolutionary radiations (Joy, 2013). The relevance of microbial symbiosis to insect behavior has emerged as a major research focus, with studies revealing that some insects exhibit sophisticated behaviors for vertical transmission of symbionts and that symbiont-induced behavioral changes are more common than previously recognized (Hosokawa and Fukatsu, 2020). Insect-fungus associations contribute fundamentally to insect physiology, ecological performance and evolutionary success by facilitating nutrient acquisition, digestion of recalcitrant substrates and detoxification of plant secondary metabolites. At the ecological level, insect–fungus partnerships mediate resource exploitation, habitat colonization, and interspecific interactions, often leading to restructuring of entire communities. Our synthesis of currently documented interactions between insects and fungi has revealed that these associations have evolved multiple times independently and are predominantly in the phyla Ascomycota (21 origins) and Basidiomycota (12 origins), These associations span across six orders of insects: Coleoptera (16 origins), Hymenoptera (7 origins), Diptera (*>*3 origins), Hemiptera (4 origins), Blattodea (1 origin) and Lepidoptera (1 origin) indicating repeated and convergent evolution of insect–fungus partnerships. Collectively, these insect-fungal associations encompass a spectrum of ecological relationships, including mutualism, commensalism, and parasitism (particularly entomopathogenicity). Furthermore, our analysis has shown that these associations are dynamic and often reflect the shared phylogenetic histories of the insect and fungal partners.

Among these interactions, mutualism emerges as the most predominant and ecologically significant association. These relationships, enabled by the unique characteristics of the partner organisms, are underpinned by a delicate balance of complementary functional traits that facilitate reciprocal benefits. Chemical signals, including volatile organic compounds, semiochemicals, and secondary metabolites, are central mediators of insect–fungal interactions, enabling host location, recognition, and behavioral modulation (Boucias et al., 2012). Odour signals relevant to beetles in deadwood habitats, including fungal volatiles and tree-derived compounds, play a critical role in mediating insect orientation, host selection, and reproductive behavior in bark beetle-fungus systems (Holighaus, 2012). Harnessing insect–microbe chemical communication presents a promising strategy for controlling insect pests in agricultural systems, with recent examples of novel insect–fungus relationships revealing semiochemicals that could be exploited for sustainable pest management (Beck and Vannette, 2017). These chemical signals have significant implications for insect olfactory systems, with fungal volatile compounds shaping host-seeking and avoidance behaviors (Ai et al., 2022). Chemical ecology in insect-microbe interactions in the neotropics reveals diverse strategies including chemical defense, communication, habitat colonization, and resource exploitation, often structuring entire communities (Menegatti et al., 2021). In tripartite insect–fungus–plant systems, nitrogen availability can significantly alter the outcome of these interactions, with cascading effects on host plant fitness (Hatcher et al., 1997a; Hatcher et al., 1997b). Unique interactions and endgames between entomopathogenic fungi and their arthropod hosts, including behavioral manipulation and host gigantism, represent some of the most highly coevolved systems in nature (Roy et al., 2006). The evolution and phylogeny of behavioral manipulation of insect hosts by parasites, including fungi, can be elucidated through experimental and phylogenetic approaches that reveal the evolutionary history of the interacting partners (Poulin, 1998). The evolutionary ecology of parasitic fungi and their host insects is characterized by rapid reciprocal adaptations, with recent studies revealing sophisticated molecular arms races between fungal virulence factors and insect immune responses (Vilcinskas, 2019). The genus Entomophthora has entered the twenty-first century, with recent research proposing six major directions for future studies, including molecular mechanisms of host manipulation, infection dynamics, and the ecological roles of these insect destroyers (Elya and De Fine Licht, 2021). The insect–fungus arms race, shaped by millions of years of coevolution, continues to reveal sophisticated molecular strategies on both sides (Leal, 2015). The parasite-host system as exemplified by interactions between entomopathogenic fungi and insects involves multiple factors including fungal virulence, host immune defense, and environmental conditions that collectively determine infection outcomes (Augustyniuk-Kram, 2011).

The evolution of mutualism necessitates shared interactions in which one species provides a service that the other species is either unable to perform or performs less efficiently. Several limitations of this systematic review should be acknowledged. First, our dataset represents a fraction of the true diversity of insect–fungus associations, which likely numbers in the thousands. Second, taxonomic and geographic biases in the literature may skew our findings toward well-studied systems (e.g., bark beetles, fungus-farming ants). Third, mycophagy was treated as a feeding behavior rather than an interaction type, potentially underrepresenting the ecological significance of fungivory. Fourth, the dataset reflects publication bias, with positive and significant results more likely to be published than negative or neutral findings. Our synthesis reveals that insect–fungal associations are evolutionarily dynamic and ecologically diverse.

However, the patterns we observe, particularly the apparent predominance of Sordariomycetes and the prevalence of parasitism, should be interpreted with caution. These apparent dominances may reflect research biases toward charismatic or economically important systems rather than genuine ecological prevalence. For instance, the high frequency of parasitism in our dataset likely overrepresents entomopathogenic interactions, reflecting the intense research interest in biological control applications. Similarly, the prominence of Coleoptera in mutualistic associations may reflect the extensive study of bark beetle–fungus systems, driven by their economic impact on forestry. Conversely, understudied groups such as Harpellomycetes (gut commensals of aquatic insects) and Laboulbeniomycetes (ectoparasites) appear underrepresented, not because they are ecologically rare, but because they have received less research attention. These sampling artifacts highlight the need for more balanced, hypothesis-driven field studies that move beyond charismatic or economically important systems. The true ecological diversity of insect–fungus associations is likely far greater than current literature suggests, and future research should prioritize understudied taxa, geographic regions, and interaction types to redress these biases.

Genomic evidence from beetle-associated *Fusarium* symbionts supports this view, demonstrating drastic genome reductions in symbiotic lineages and multiple independent acquisitions of fungal symbionts by tortoise beetles (Pitsillides et al., 2025). Beyond enzymatic convergence, insect–fungus interactions are shaped by a co-evolutionary molecular arms race between host immune defenses and fungal counter-adaptations. Insects have evolved multiple layers of defense against fungal invasion, including physical barriers (cuticle thickening), biochemical responses (antimicrobial peptides, melanization, and hemocyte-mediated phagocytosis), and behavioral strategies (grooming and avoidance). In response, fungi have developed sophisticated counter-mechanisms: cuticle-degrading proteases and chitinases, immunosuppressive secondary metabolites (e.g., oosporein, destruxins), inhibitors of host melanization, and even behavioral manipulation of the host to enhance spore dispersal. These reciprocal adaptations are summarized in **Table 2**. Particularly, the evolutionary transition from free-living saprotrophs to obligate symbionts or specialized endoparasites has consistently selected for these virulence factors, suggesting that the molecular toolkit for host exploitation evolved repeatedly through convergent evolution. The transition to mutualism, in contrast, required the loss or attenuation of these virulence traits, coupled with the acquisition of nutritional provisioning capabilities (e.g., CAZyme expansions for lignocellulose degradation). This dichotomy, virulence in pathogens versus nutritional mutualism in symbionts, underscores the plasticity of insect–fungus associations and highlights the importance of context-dependent gene regulation in shaping interaction outcomes. **Table 2** summarizes the molecular arms race between host immune defenses and fungal counter-adaptations during insect–fungus interactions.

**Table 2:** Molecular arms race in insect–fungus interactions: host immune defenses and fungal counter-adaptations.

Host defense mechanism	Fungal counter-adaptation	Example system	References
Cuticle thickening (physical barrier)	Cuticle-degrading enzymes (proteases, chitinases)	*Beauveria bassiana* vs. *Galleria mellonella*	Vilcinskas, 2019; Shang et al., 2015
Antimicrobial peptide production (e.g., defensins, cecropins)	Suppression of host immune gene expression	Metarhizium robertsii vs. Drosophila melanogaster	Wang et al., 2017; Dubovskiy, 2021
Melanization and encapsulation	Evasion of melanization; production of melanization inhibitors	*Ophiocordyceps unilateralis* vs. *Camponotus* ants	Andersen et al., 2009; Araújo and Hughes, 2016
Hemocyte-mediated phagocytosis	Secretion of immunosuppressive toxins (e.g., oosporein, destruxins)	*Beauveria bassiana*, *Metarhizium* spp.	Nicoletti and Becchimanzi, 2022; Litwin et al., 2020
Activation of phenoloxidase cascade	Inhibition of phenoloxidase activity	*Entomophthora muscae* vs. houseflies	Elya and De Fine Licht, 2021
Behavioral resistance (e.g., grooming, avoidance)	Behavioral manipulation (e.g., “summit disease, “ “death grip”)	*Ophiocordyceps* spp., *Entomophthora* spp.	Roy et al., 2006; Elya and De Fine Licht, 2021
Detoxification of fungal toxins	Evolution of toxin resistance/tolerance	*Drosophila melanogaster* vs. *Aspergillus* molds	Trienens and Rohlfs, 2011

Insects and fungi, though both heterotrophs, differ in mobility, defense mechanisms, and nutrition. Insects are fast-moving and possess an advanced immune and digestive system. In contrast, fungi are mostly sedentary and use secondary metabolites to protect against stresses and digest food using extracellular enzymes. These complementary characteristics, carefully balanced in the mutualistic relationship developed over the years, are believed to be the reasons for the successful growth and survival of insects and fungi (Biedermann and Vega, 2020).

Fungal taxa within the classes Sordariomycetes and Entomophthoromycetes are among the most prevalent insect-associated fungal lineages in natural ecosystems, with a significant proportion of them having entomopathogenic lifestyles. Members of the order Entomophthorales such as *Furia, Conidiobolus, Entomophaga*, and *Erynia* are among the most virulent insect pathogens documented often causing rapid epizootics in host populations. However, despite their potent insecticidal activity, their practical use as biopesticide preparations is constrained by technical challenges associated with culturing them under laboratory conditions and for mass production. However, entomopathogenic fungi within the class Sordariomycetes (phylum Ascomycota), many of which are facultative saprotrophs, have been more successfully developed as commercial biopesticide formulations thanks to their relative ease of cultivation in the laboratory and scalability (Mascarin and Jaronski, 2016). Notably, genera such as *Metarhizium*, *Beauveria*, *Paecilomyces*, *Isaria*, and *Lecanicillium* are widely exploited as biopesticides due to their high insecticidal effectiveness. These fungi exhibit a broad spectrum of activity, enabling them to infect a diverse range of arthropod species and represent sustainable alternatives to synthetic chemical pesticides and thus contribute to reduced environmental contamination that has been associated with the overreliance on chemical pesticides (Khan et al., 2012; Orduño-Cruz et al., 2016; Ríos-Moreno et al., 2016).

Moreover, fungal entomopathogens are heterogeneous organisms with diverse ecological roles beyond serving as insect pathogens. Species of the genera *Metarhizium* and *Beauveria* are common soil fungi that not only control insect populations but also establish intricate associations with plants (Rizal et al., 2024, 2026). These fungi often form endophytic associations colonizing plant roots, stems, and leaves where they confer indirect protection against herbivorous insects and, in some cases, phytopathogens. Recent research has revealed that endophytic entomopathogenic fungi can enhance plant resistance through multiple mechanisms beyond direct pathogenicity. Aravinthraju et al. (2024) comprehensively reviewed how endophytic entomopathogenic fungi alter plant secondary metabolites, volatile organic compounds, and microbiomes, thereby enhancing plant resistance to pests and diseases. These fungi also exhibit compatibility with other pest management strategies, including predators, parasitoids, and chemical inputs. Beyond direct pathogenicity, insect–fungus interactions can indirectly affect host plant fitness; for example, fungal infection of insect herbivores can reduce herbivory pressure, while fungal endophytes may alter plant growth and overwintering success (Hatcher, 1996). Endophytic insect-pathogenic fungi have diverse biology and applications, functioning as plant endophytes while simultaneously providing defense against insect herbivores thus offering dual benefits for crop protection (Branine et al., 2019).

However, translating endophytic entomopathogenic fungi from laboratory to field applications poses several challenges. Panwar and Szczepaniec (2024) synthesized current knowledge on the effectiveness of endophytic *Beauveria* and *Metarhizium* against diverse insect pests and highlighted that field efficacy can be variable and influenced by environmental factors, fungal strain selection, and plant genotype. Addressing these constraints requires further research on formulation stability, application methods, and integration with other pest management tactics.

Mycopathogens of soil insects play a crucial role in natural pest suppression, and understanding their ecology and diversity is essential for harnessing them in biological control programs (Keller and Zimmermann, 1989). Altered gene expression in the parasitoid *Pteromalus puparum* after infection with the entomopathogenic fungus *Beauveria bassiana* reveals molecular mechanisms underlying immune responses and contributes to our understanding of insect-fungus interactions (Yang et al., 2023). At the molecular level, the success of some entomopathogens is underpinned by complex regulatory mechanisms some of which have been characterised. For example, DNA methyltransferases in the entomopathogenic fungus *Metarhizium robertsii* contribute to fungal development, stress tolerance, and virulence (Wang et al., 2017). Recent advances in genetic engineering have further enhanced the pathogenicity, host range, and environmental adaptability of entomopathogenic fungi. Ahmad et al. (2025) comprehensively reviewed genetic modifications in *Metarhizium anisopliae*, *Purpureocillium lilacinum*, and *Beauveria bassiana*, highlighting CRISPR-Cas9 applications for precise gene editing to improve virulence and stress tolerance. Such approaches offer promising avenues for developing more effective mycoinsecticides with enhanced field performance.

The insect fungus *Metarhizium robertsii* is a rare cause of keratitis in humans, demonstrating that entomopathogenic fungi can occasionally cause opportunistic infections in mammals and highlighting the importance of understanding cross-kingdom interactions (Palazzolo et al., 2022). Chlorimuron ethyl has been developed as a new selectable marker for disrupting genes in the insect-pathogenic fungus *Metarhizium robertsii*, facilitating functional genomic studies of fungus-insect and fungus-plant interactions (Lin et al., 2011). Carbohydrate storage in the entomopathogenic fungus *Beauveria bassiana* plays a crucial role in its survival, virulence, and commercial formulation for biological control (Bidochka et al., 1990). Beyond direct pathogenicity, insect–fungus interactions can indirectly affect host plant fitness. These fungi are endophytes that colonize plant roots, stems, and leaves (Jaber and Enkerli, 2017). Intriguingly, experimental evidence has shown that species such as *Metarhizium robertsii* and *Beauveria bassiana* supply insect-derived nitrogen to host plants during the parasitism, thereby enhancing plant growth and productivity (Behie and Bidochka, 2014; Ríos-Moreno et al., 2016). Furthermore, *Beauveria bassiana* functions as an endophyte in approximately 25 plant species, aiding in managing pests and fungal pathogens affecting plants (McKinnon et al., 2017; Vega, 2018).

Insect-fungal interactions that exhibit commensalism primarily involve microbial interactions within the insect gut, from which the host insect does not derive any direct benefits. However, in certain associations, such as those involving Drosophila, yeast-mediated fermentation enhances nutrient utilization and thus directly influences host development and fitness, indicating that even seemingly neutral associations may have subtle functional consequences. These interactions are of immense ecological importance, as they help maintain the delicate balance of our ecosystems. They can also be used to bolster populations of beneficial insects, such as bees and ladybugs, which are crucial to sustainable agriculture. By introducing beneficial fungi to these key insects, we can create a synergistic effect that enhances the quality of life for these essential species and contributes to the overall success of agricultural systems.

Symbiotic associations between insects and fungi constitute some of the most intricate and significant biological interactions, shaping key processes for both partners. Increasing evidence suggests that these associations keep evolving rapidly, with some transitioning from pathogenic to mutualistic and vice versa or expanding into entirely new ecological niches. Indeed, there is every likelihood that there exist numerous dimensions of some of these associations that are yet to be characterized. For example, recent studies continue to reveal novel dimensions of insect–fungus symbiosis. Mineral acquisition strategies, such as iron uptake in fungi like *Penicillium herquei* reveals that mineral elements play a previously unrecognized role in insect–fungus symbiosis. Emerging associations, such as those between the insect *Leptoglossus occidentalis* and the fungal pathogen *Diplodia pinea*, demonstrate how environmental change can facilitate the formation of novel ecological interactions. Additionally, multitrophic systems involving aphids, their insect and fungal natural enemies, and host plants highlight the complexity of ecological networks that underpin biological control strategies going beyond pairwise interactions (Qiu et al., 2025; Luchi et al., 2012).

Collectively, these examples illustrate that insect–fungus interactions rarely occur in isolation. Tri-trophic dynamics involving plants, fungi, and insects create complex feedback loops that can amplify or dampen the effects of individual interactions. For instance, fungal endophytes can prime plant defenses against herbivores, while herbivory can in turn influence endophyte colonization and secondary metabolite production. Similarly, entomopathogenic fungi can indirectly benefit plants by reducing herbivore pressure, while simultaneously competing with other soil microbes for resources. Understanding these multi-trophic feedbacks is essential for predicting the outcomes of insect–fungus associations in natural and agricultural systems, and for developing effective biocontrol strategies that account for community-level interactions.

Fungal interactions with other pests, including insects and mites, have significant and complex effects on agroecosystems, influencing nutrient cycling, pest population dynamics, and crop health (David and Slot, 2022). Furthermore, insect interactions with mycotoxin-producing fungi have important implications for both natural ecosystems and agricultural systems, affecting host plant health, food safety, and livestock (Dowd, 2024). Tri-kingdom interactions among insects, plants, and fungi in shifting environments can explain host susceptibility and insect-plant host shifts, with important implications for understanding species interactions under environmental change (Morris, 2025). Understanding these principles is not only fascinating but also crucial for our future research and discoveries. The focus on insect-fungal interactions forged over millions of years of coevolution is increasingly focusing beyond basic biological inquiry to critical areas of practical application and innovation, such as in agriculture, biotechnology, and medicine. For example, entomopathogenic fungi, particularly those in the *Beauveria* and *Metarhizium* genera, have gained worldwide recognition as potential eco-friendly alternatives to chemical pesticides, with current efforts focused on enhancing their efficiency through genetic modification. Moreover, insect-fungal symbiosis systems, such as those involving ambrosia beetles and fungi-farming termites, harbor highly efficient lignocellulose-degrading microbial communities. Scientists are focusing on how such systems could be used to break down complex pollutants, including synthetic plastics. Studies on behavior-manipulating fungi, such as *Ophiocordyceps* and *Entomophthora*, offer valuable insights and potential for developing novel insect behavioral modulation strategies that could find practical application in targeted pest management, thereby reducing overreliance on synthetic chemical pesticides and shifting toward more sustainable agricultural practices.

## 6 Conclusion

Insect–fungal interactions constitute a complex evolutionary and ecological association that spans a continuum from mutualism to parasitism, from facultative to obligate, often extending across multiple trophic levels. As highlighted throughout this systematic review, these associations, once regarded as simple occurrences of nature, have been shown to be highly dynamic, influencing a wide range of community structures, biological organizations, and processes. Our synthesis suggests that these associations are more prevalent and diverse than previously recognized, with documented interactions across multiple insect orders and fungal classes. However, the true extent of insect–fungus associations remain unknown, and the patterns described here should be viewed as trends within the compiled literature rather than definitive ecological rankings. Several critical knowledge gaps remain, including the undercharacterized functional diversity of insect-associated fungal communities, the molecular mechanisms underlying host specificity, and the impacts of climate change on interaction outcomes. Most interactions have been studied in isolation, limiting understanding of community-level effects and the complex dynamics of multi-partner systems.

Currently, there is a renaissance of interest in insect–fungus associations, driven by advances in integrative approaches spanning molecular biology, genomics, metabolomics, chemical ecology, and systems biology. This integrative approach continues to unravel the intricacies of the hidden diversity and functionality of these interactions. Significantly, more recent research has shifted from a predominantly descriptive biological perspective to a more translational, application-oriented approach. Therefore, while earlier research focused mostly on ecological interactions, diversity, and life history, emerging research is increasingly focused on harnessing these systems for practical applications. In the context of sustainable agriculture, insect–fungus associations offer potential opportunities to develop eco-friendly biological pest control strategies as alternatives to synthetic pesticides, although field efficacy remains variable and formulation challenges persist. In biotechnology, there is a growing interest in exploring these associations to discover novel enzymes with diverse industrial applications. Insect-associated fungi have been shown to possess metabolic capabilities for producing lignocellulolytic, chitinolytic, and detoxification enzymes. Furthermore, these systems are increasingly recognized as potential sources of novel bioactive and lead compounds with pharmaceutical relevance. However, translating these findings into practical applications requires overcoming significant challenges related to commercialization, registration, and field performance.

### 6.1 Knowledge gaps and future directions

Based on our systematic synthesis, we identify the following critical knowledge gaps requiring future research:

1. Functional diversity of insect-associated fungal communities: While taxonomic diversity is increasingly well-characterized, the functional roles of most insect-associated fungi remain unknown. Metagenomic and metatranscriptomic approaches are needed to elucidate the functional capabilities of these communities.
2. Molecular mechanisms of host specificity: The genetic and biochemical basis of host specificity in insect–fungus interactions are poorly understood. Comparative genomics and functional studies are needed to identify key determinants of host range.
3. Climate change impacts: How changing environmental conditions will affect the outcomes of insect–fungus interactions remain largely unexplored. Temperature sensitivity, altered phenology, and range shifts may disrupt established associations.
4. Multi-partner interactions: Most studies focus on pairwise interactions, yet insect–fungus associations often involve additional partners, including bacteria, other fungi, and host plants. Understanding these complex networks is essential for predicting ecological outcomes.
5. Translational research: Bridging the gap between laboratory findings and field applications requires improved formulations, delivery methods, and integration with existing pest management strategies. **Figure 7** summarizes the integrative approaches driving current advances in insect–fungus research, along with key knowledge gaps requiring future investigation.

**Figure 7:**
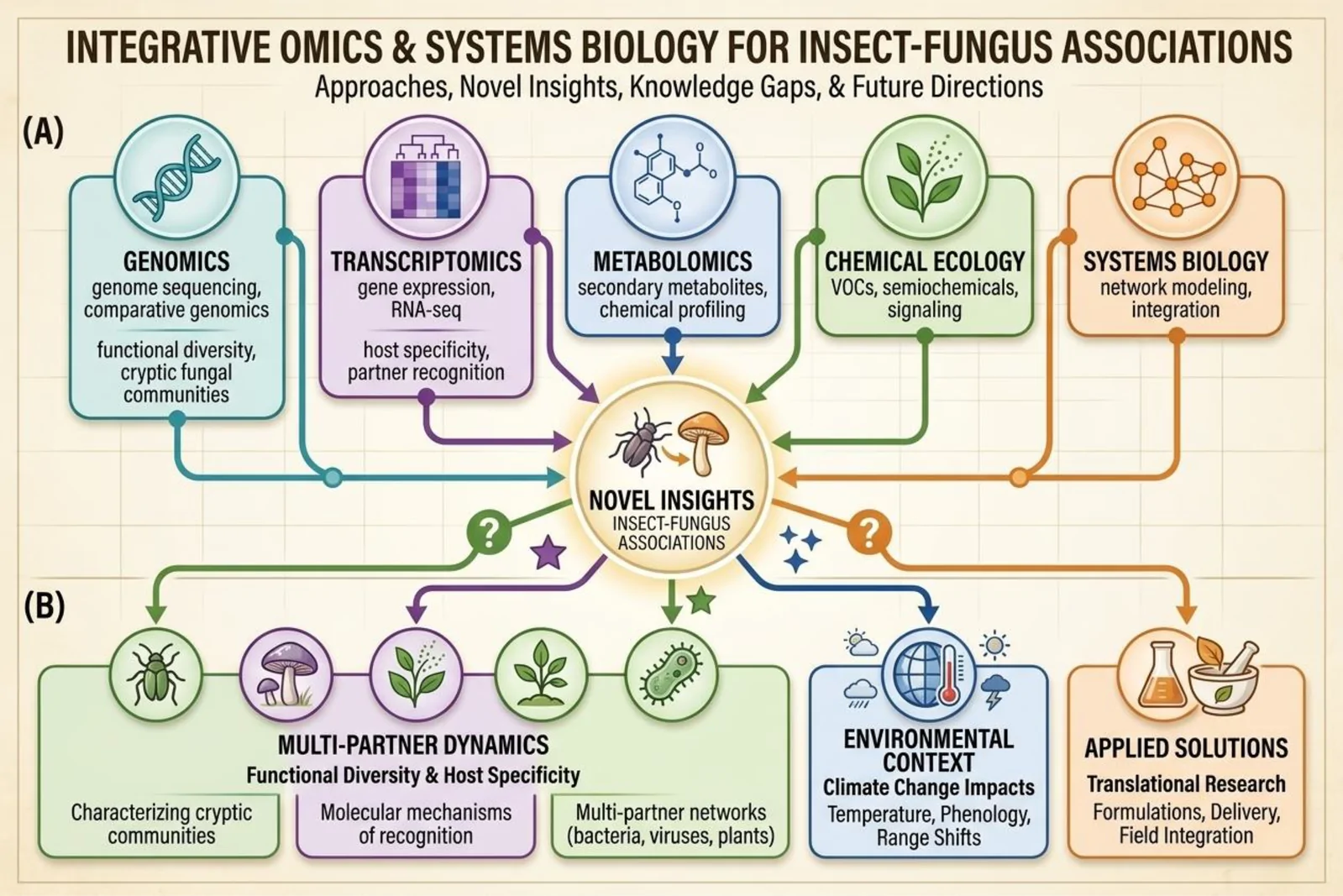
Integrative approaches and future directions in insect–fungus research. **(A)** Multi-omics and systems biology approaches (genomics, transcriptomics, metabolomics, chemical ecology, systems biology) converge to generate novel insights into insect–fungus associations. **(B)** Key knowledge gaps: functional diversity, host specificity, climate change impacts, multi-partner networks, and translational research (Created with BioRender).
